# Association of JAK/STAT genetic variants with cutaneous melanoma

**DOI:** 10.3389/fonc.2022.943483

**Published:** 2022-08-02

**Authors:** Gabriela Vilas Bôas Gomez, Gustavo Jacob Lourenço, Lummy Maria Oliveira Monteiro, Rafael Silva Rocha, Kimberly Anne McGrail Fernández, Juan Angel Recio, Caroline Torricelli, Lilian Oliveira Coser, Alexandre Leite Rodrigues Oliveira, Juliana Carron, Aparecida Machado Moraes, Carmen Silvia Passos Lima

**Affiliations:** ^1^ Laboratory of Cancer Genetics, Faculty of Medical Sciences, University of Campinas, Campinas, São Paulo, Brazil; ^2^ Department of Cellular and Molecular Biology, Faculty of Medicine of Ribeirão Preto, University of São Paulo, São Paulo, Brazil; ^3^ Animal Models and Cancer Laboratory, Vall d’Hebron Research Institute, Hospital Universitari Vall d’Hebron, Barcelona, Spain; ^4^ Laboratory of Nerve Regeneration, Department of Structural and Functional Biology, Institute of Biology, University of Campinas, São Paulo, Brazil; ^5^ Department of Anesthesiology, Oncology and Radiology, Faculty of Medical Sciences, University of Campinas, São Paulo, Brazil

**Keywords:** cutaneous melanoma, *JAK1*, *JAK2*, *STAT3*, genetic variant, risk, prognosis

## Abstract

**Background:**

The Janus-activated kinase (JAK)-signal transducer and activator of transcription (STAT) signaling pathway regulates cutaneous melanoma (CM) development and progression. The JAK1, JAK2, and STAT3 proteins are encoded by polymorphic genes. This study aimed to verify whether single-nucleotide variants (SNVs) in *JAK1* (c.1648+1272G>A, c.991-27C>T), *JAK2* (c.-1132G>T, c.-139G>A), and *STAT3* (c.*1671T>C, c.-1937C>G) altered the risk, clinicopathological aspects, and survival of CM patients as well as protein activity.

**Methods:**

CM patients (*N* = 248) and controls (*N* = 274) were enrolled in this study. Genotyping was performed by real-time polymerase chain reaction (PCR), and *JAK1*, *JAK2*, and *STAT3* expression was assessed by quantitative PCR (qPCR). *STAT3* c.-1937C>G SNV was investigated by luciferase, qPCR, western blot, apoptosis, and cell cycle assays in SKMEL-28 cells with CC or GG genotype.

**Results:**

Individuals with *STAT3* c.*1671TT and c.-1937CC genotypes and TC haplotype of both SNVs were under about 2.0-fold increased risk of CM. Specific *JAK1*, *JAK2*, and *STAT3* combined genotypes were associated with up to 4.0-fold increased risk of CM. Higher luciferase activity [4,013.34 vs. 2,463.32 arbitrary units (AU); *p* = 0.004], *STAT3* expression by qPCR (649.20 *vs*. 0.03 AU; *p* = 0.003) and western blot (1.69 *vs*. 1.16 AU; *p* = 0.01), and percentage of cells in the S phase of the cell cycle (57.54 vs. 30.73%; *p* = 0.04) were more frequent in SKMEL-28 with *STAT3* c.-1937CC than with GG genotype. CM cell line with CC genotype presented higher STAT3 protein levels than the one with GG genotype (1.93 *versus* 1.27 AU, *p* = 0.0027).

**Conclusion:**

Our data present preliminary evidence that inherited abnormalities in the JAK/STAT pathway can be used to identify individuals at a high risk of CM, who deserve additional attention for tumor prevention and early detection.

## Introduction

Janus-activated kinase (JAK)-signal transducer and activator of transcription (STAT) has been identified as an important signaling pathway involved in cell proliferation, mainly through JAK1, JAK2, and STAT3 proteins, mediating various biological responses induced by cytokines and growth factors (1). In regular melanocytes, the activation of JAK/STAT is rapid and transient, but growth factors and cytokines secreted by abnormal cells as well as in the tumor microenvironment keep this pathway always activated (2, 3). Besides its role in cell proliferation, the JAK/STAT pathway induces angiogenesis, inhibits apoptosis and immune system response, and promotes metastasis, resulting in cutaneous melanoma (CM) development and progression (2–4).

Activation of *STAT3* has been noted as an important event in melanoma progression and metastasis. In addition, JAK1, JAK2, and STAT3 levels have altered the prognosis of CM patients (2, 3). Due to this, targeting STAT3 has been considered a potential therapeutic strategy for CM treatment since several STAT3 inhibitors revealed promising results in early-phase clinical trials (1, 5). It is already well known that JAK1, JAK2, and STAT3 proteins are encoded by polymorphic genes (6). Thus, healthy individuals may present distinct CM risks; CM patients treated equivalently may present diverse outcomes as well.

*JAK1* c.1648+1272G>A (rs310211) single-nucleotide variant (SNV) is characterized by a G>A substitution in the intronic region 117268, and *JAK1* c.991-27C>T (rs2256298) SNV consists of the exchange of a C>T in the intronic region 106506, both in the splicing region of the gene (7–9). *JAK2* c.-1132G>T (rs1887429) and c.-139G>A (rs2274472) SNVs are characterized by G>T and G>A modifications in the gene promoter region, respectively, and that gain in the binding site of transcription factors may lead to changes in protein production (10–12). A T>C change in the 3′-UTR region of *STAT3* characterizes the c.*1671T>C (rs1053004) SNV. The gene binding efficiency of microRNAs 423-5p, 31-5p, 21-5p, and 99b-3p is low in carriers of the allele “C”, which leads to a decrease in STAT3 mRNA and protein levels (12–14). Lastly, the *STAT3* c.-1937C>G (rs4796793) SNV is characterized by an exchange of a C>G in the gene promoter region (15). The allele “G” was associated with a lower STAT3 expression in B lymphocyte cell lines and better response of renal cell carcinoma patients to interferon alpha (IFNα) than the allele “C” (16). Melanoma cells carrying the allele “G” were the most sensitive to IFNα *in vitro* but did not predict IFNα efficacy in CM patients (15). Thus, the mechanism by which the SNVs interfere in protein function is not well defined.

We analyzed in the present study, for the first time, associations of *JAK1* (c.1648+1272G>A, c.991-27C>T), *JAK2* (c.-1132G>T, c.-139G>A), and *STAT3* (c.*1671T>C, c.-1937C>G) SNVs with CM risk, clinicopathological aspects, and prognosis and conducted functional studies to understand their biological consequences.

## Materials and methods

### Study population

The study included 248 consecutive patients with a median age of 55 years and primary CM attended at diagnosis at the General Hospital of the University of Campinas between January 2001 and May 2018. Patients with acral or amelanotic tumor were excluded from the analyses because they present distinct histological, phenotypic, and genetic characteristics, suggesting a biological difference when compared to other types of CM. The control group comprised of 274 blood donors with a median age of 50 years seen at the Hematology and Hemotherapy Center of the same university and in the same period. All procedures involving patients and controls were carried out according to the Helsinki Declaration, and the study was approved by the local research ethics committee (process 58186316.1.0000.5404).

### Data collection

A standardized questionnaire was applied in patients and controls to obtain clinical information, such as age, gender, presence of nevi, phototype, sun exposure, previous sunburns, and type of sun exposure. Phototype was classified following the classification of Fitzpatrick (17). Six skin phototypes are possible: I—individuals with light white skin, are very sensitive to the sun, and who never tan; II—individuals with white skin who always burn and tan; III—individuals with light brown skin who burn and tan moderately; IV—individuals with dark brown skin who burn little and always tan; V—individuals with brown skin that rarely burn and who always tan; and VI—individuals with black skin who never burn and always tan (17). Sun exposure was defined as intermittent for individuals who were exposed to the sun for more than 2 h a day for more than 10 years and related to recreational activities by less than 50% on the week or vacation. Sun exposure was defined as chronic for those individuals who performed work or home activities more than 50% of the time under exposure. Individuals not exposed to the sun were the ones who did not fit in the previous definitions (18, 19).

The tumor pathologic aspects and survival data of CM patients were obtained from the patients’ medical records. CM diagnosis was established by histopathological evaluation of tumor fragments embedded in paraffin and stained with hematoxylin and eosin (20). The tumor was measured by Breslow thickness (21) and Clark levels (22), and clinical stage was defined by the 7th American Joint Committee on Cancer criteria (23). The patients were conventionally treated as described in previous studies with the same population (24). In summary, patients with localized tumor were submitted to excisional surgery, and lymphadenectomy was performed in those with clinically positive lymph nodes or the ones with histological tumor infiltration. Patients with a single operable metastasis or recurrence were treated by surgical resection, whereas those patients with inoperable recurrence or multiple metastases received chemotherapy with dacarbazine. Radiotherapy has been used in patients with hemorrhagic lesions and bone or brain metastases.

### SNV selection for study

All steps used for selecting SNVs for the study are presented in **Supplementary Figure S1**. The entire sequences of *JAK1*, *JAK2*, and *STAT3* genes were obtained from dbSNP database (http://www.ncbi.nlm.nih.gov/projects/SNP). First, SNVs that have previously been associated with the risk/survival of cancer patients were selected from public databases (*N* = 49). Subsequently, the SNVs mostly studied in different types of cancer and that present minor allele frequency ≥0.10 in the HapMap global population were selected (*N* = 20) (**Supplementary Table S1**). Finally, *in silico* analyses with Variant Effect Predictor (25), Human Splicing Finder (26), Genomatrix (27), SNPinfo (28), MicroSNiPer (29), and MirSNPscore (30) programs were performed. Two SNVs of each gene were selected for the study to analyze the pathway as a whole as well as SNVs with higher biological plausibility of being involved in tumor origin or progression (*N* = 6) (**Supplementary Table S2**).

*JAK1* c.1648+1272G>A (rs310211), *JAK1* c.991-27C>T (rs2256298), *JAK2* c.-1132G>T (rs1887429), *JAK2* c.-139G>A (rs2274472), *STAT3* c.*1671T>C (rs1053004), and *STAT3* c.-1937C>G (rs4796793) were selected for the analyses of CM risk (comparisons of genotype frequencies in patients and controls), clinicopathological aspects (comparisons of genotype frequencies only in groups of patients), patients’ survival, and gene expression. Thus, *STAT3* c.-1937C>G was the SNV of greatest interest in the study and the object of additional functional analyses.

### Cell line selection for study

Fourteen CM cell lines were analyzed in this study: G361, A375, SKMEL-28, SKMEL-103, MSK8, MMLN9, MMLN10, MMLN14, MMLN23, MMGP3, MMSK22, MMLN24, UACC903, and MEWO. SKMEL-28 was obtained from a Rio de Janeiro cell bank (Rio de Janeiro, Brazil) with short tandem repeat analysis, and the remaining cell lines were acquired from the Animal Models and Cancer Laboratory of Vall d’Hebron Research Institute (Barcelona, Spain). All cell lines were obtained from human melanoma and tested for *BRAF* and *NRAS* drive mutations and *STAT3*c.-1937C>G genotypes.

To obtain melanoma cells with the same characteristics and genetic profile but expressing the wild-type or variant genotypes of *STAT3* c.-1937C>G SNV for the functional studies, the genetic transformation of the SKMEL-28 cells was performed. These cells were chosen for study due to its well-known molecular characterization (*BRAF* mutated), ease of cultivation, and easy genetic transformation. Thus, SKMEL-28 cells with *STAT3* c.-1937CC and GG genotype were used in luciferase, qPCR, apoptosis, cell cycle, and western blot assays. The modified SKMEL-28 and non-modified cell lines, characterized by *RAS* and *BRAF* mutations and *STAT3* c.-1937C>G genotypes, were selected for the analyses of *STAT3* levels; unmodified cell lines were included in the determination of STAT3 levels with the purpose of increasing the cell line sample and verifying, through comparison with modified SKMEL-28 cells, whether STAT3 levels could be altered by the genetic modification of cells.

### Cell line culture

Cell lines (2.5 × 10^6^/plate) were cultured in 60-mm-diameter plates containing a specific culture medium for each cell type: Dulbecco’s modified Eagle’s medium (DMEM) (Gibco, USA) supplemented with 10% fetal bovine serum (FBS) for G361, A375, SKMEL-28, and SKMEL-103 cell lines; DMEM (Gibco, USA) supplemented with 20% FBS for MSK8, MMLN9, MMLN10, MMLN14, MMLN23, MMGP3, MMSK22, and MMLN24 lines; RPMI medium (Gibco, USA) supplemented with 10% FBS for UACC903 line; and EMEM medium (Gibco, USA) supplemented with 10% FBS for MEWO line. Subsequently, 1% penicillin–streptomycin (100 U/ml) (Gibco, USA), 1% L-glutamine (Gibco, USA), and 100 μl plasmocin (Gibco, USA) were added to each plate. The cells were grown at 37°C in 5% CO_2_ condition and tested for mycoplasma contamination. All experiments were performed using mycoplasma-free cells (31).

### Genotyping

Genomic DNA was obtained from the leukocytes of peripheral blood samples of patients and controls and from all melanoma cell lines.

Genotyping of individuals and all cell lines was performed by real-time polymerase chain reaction (PCR) method using TaqMan SNV genotyping assay [Applied Biosystems, USA; *JAK1* c.1648+1272G>A (C_176626140), *JAK1* c.991-27C>T (C_176627520), *JAK2* c.-1132G>T (C_1209582910), *JAK2* c.-139G>A (C_1618193310), *STAT3* c.*1671T>C (C_17952851) and *STAT3* c.-1937C>G (C_2797721310)] according to the manufacturer’s instructions. Positive and negative controls were used in all reactions. For quality control purposes, 15% of the samples were genotyped twice with a 100% concordance rate.

### *JAK1*, *JAK2*, and *STAT3* expression in peripheral blood

Gene expression was analyzed by quantitative PCR (qPCR). Total RNAs from the leukocytes of the peripheral blood of 40 CM patients and 60 controls with distinct genotypes of *JAK1* (c.1648+1272G>A, c.991-27C>T), *JAK2* (c.-1132G>T, c.-139G>A), and *STAT3* (c.*1671T>C, c.-1937C>G) SNVs were extracted with Trizol reagent (Life Technologies, USA) according to the manufacturer’s instructions. RNAs were selected based on integrity and availability to carry out the technique, and all individuals who met these criteria were evaluated by qPCR. cDNA was generated using SuperScript III reagents (Life Technologies, USA), and the experiments were performed with SYBR Green PCR Master Mix reagents (Applied Biosystems, USA). The relative expression level was normalized by β-actin reference gene with the 2^-DDCt^ cycle threshold method. Forward and reverse specific primers in duplicate per sample and a negative control without template were included in each plate (**Supplementary Table S3**). The experiments (15%) were repeated with 100% agreement, and the results were expressed in arbitrary units (AUs).

### *STAT3* promoter region activity in modified SKMEL-28 cell line

The *STAT3* promoter region activity was analyzed in SKMEL-28 cells with CC or GG genotype of *STAT3* c.-1937C>G SNV by dual luciferase reporter assay. For cell transformation, forward and reverse primers (**Supplementary Table S3**) were designed to amplify 1,940 base pairs of *STAT3* promoter region containing *STAT3* c.-1937C>G SNV by PCR with 2 U of Platinum™ Taq DNA Polymerase, High Fidelity (Thermo Scientific, USA). The PCR products were cloned into pGL-3 basic vector (Promega, USA) using restriction enzymes *Kpn1* and *Nco1* (Thermofisher, USA) according to standard protocols (32, 33). After these procedures, pGL-3luc_C and pGL-3luc_G plasmids were obtained and were transformed into *E. coli* DH5α-competent bacteria (Invitrogen, USA) by electroporation (32, 33). The final construction was verified by restriction enzyme digestion and sequencing by Sanger 3730xL (Applied Biosystems, USA).

SKMEL-28 cells were grown in 12-well cell culture plates (1 × 10^5^ cells/well) for 24 h. Then, they were transiently transfected with a promoterless luciferase vector (empty pGL3luc-basic) or with pGL3luc_C or pGL3luc_G plasmids, and *Renilla* luciferase control reporter (pRL) was used as the normalizing control (Promega, USA). For transfection, Lipofectamine™ 2000 (Invitrogen, USA) and reduced serum medium Opti-MEM (Gibco, USA) were used according to the manufacturer’s recommendations. The cells were harvested 24 h after transfection, and luciferase assays were performed with Dual Luciferase Assay Kit (Promega, USA) according to the manufacturer’s protocol. Relative firefly luciferase activity was normalized for the pRL vector activity. The assays were performed in triplicate with a negative control in each reaction, and the results were expressed in AUs.

### *STAT3* expression in modified SKMEL-28 cell line

*STAT3* expression was analyzed in SKMEL-28 cells with *STAT3* c.-1937CC or GG genotype by qPCR.

For cell transformation, a complete *STAT3* gene was cloned into pGL-3 basic vector (Promega, USA). For this, pGL3luc_C and pGL3luc_G plasmids were subjected to luciferase region removal by restriction enzymes (*Nco1* and *Xba1*) (Thermofisher, USA) (32, 33). Subsequently, the fragments of interest were amplified from cDNA with forward and reverse primers (**Supplementary Table S3**) and then were ligated to the vector by T4 DNA ligase enzyme (Life Technologies, USA) (32, 33). In the end, new vectors (pGL3_*STAT3*_C and pGL3_*STAT3_*G) containing the coding region until the gene stop codon were obtained. The SKMEL-28 cells were grown in 60-mm cell culture plates (2.5 × 10^6^ cells/plate) for 24 h. Then, they were transiently transfected with pGL3-basic empty or pGL3_*STAT3*_C or pGL3_*STAT3_*G vectors. For transfection, polyethylenimine (PEI) (Veritas Biotecnologia, USA) and reduced serum medium Opti-MEM (Gibco, USA) were used according to the manufacturer’s instructions. The vector with green fluorescent protein (GFP) was used in transfection monitoring. At the end of the experiment, SKMEL-28 cells with *STAT3* c.-1937CC and c.-1937GG genotypes were obtained for the analyses of STAT3 expression, apoptosis and cell cycle.

For the analysis of *STAT3* expression, the cells were harvested 24 h after transfection, and RNA extraction was performed with the Direct-zol RNA MinPrep kit (Zymo Research, USA) according to the manufacturer’s protocols. Assays were performed in triplicate using a negative control in each reaction. For each experiment, *STAT3* expression by qPCR was performed, and the results were expressed in AUs.

### *STAT3* protein levels in modified SKMEL-28 and unmodified cell lines

STAT3 protein levels were analyzed by western blot in SKMEL-28 cells transfected with the empty vector with *STAT3* c.-1937CC or GG genotype. In addition, 14 unmodified melanoma cell lines (G361, A375, SKMEL-28, SKMEL-103, MSK8, MMLN9, MMLN10, MMLN14, MMLN23, MMGP3, MMSK22, MMLN24, UACC903, and MEWO) with different *STAT3* c.-1937C>G genotypes were also used in the determination of STAT3 levels.

The cells were lysed with radioimmunoprecipitation assay buffer containing protease inhibitors. Total protein concentrations were measured by the Bradford method (1976) and bovine serum albumin standard curve. For western blot, total proteins (50 µg) were subjected to 10% sodium dodecyl sulfate polyacrylamide gel electrophoresis, and then they were transferred to nitrocellulose membranes. The membranes were blocked with 5% skimmed milk in phosphate-buffered saline (PBS-Tween) and incubated with specific primary antibodies anti-STAT3 (Santa Cruz, USA) and anti-glyceraldehyde-3-phosphate dehydrogenase (GAPDH) (Santa Cruz, USA) overnight at 4°C. A horseradish peroxidase-conjugated goat anti-mouse IgG was used as the secondary antibody (Santa Cruz, USA). ECL Western Blot Detection Reagents kit (GE Healthcare, USA) was used for protein detection, and the signal intensity was analyzed by ImageJ software (National Institutes of Health, USA). The levels of GAPDH were used as loading control. The protein levels were expressed in AUs.

### *STAT3* in apoptosis and cell cycle assays in modified SKMEL-28 cell line

For apoptosis assay, 2.5 × 10^6^ SKMEL-28 cells were cultured in 60-mm cell culture plates with DMEM supplemented with 10% FBS. On the next day, the cells were transfected with empty pGL3 or pGL3_*STAT3*_C or pGL3_*STAT3_*G vectors of the *STAT3* c.-1937C>G SNV using PEI watering system (Veritas, USA). GFP vector was used in transfection monitoring. After 6 h of transfection, the cells were subjected to 200 μM of hydrogen peroxide (H_2_O_2_) (Sigma, USA) in DMEM without FBS and cultured in an atmosphere of 5% CO_2_ at 37°C for 22 h to induce apoptosis. A cell culture without H_2_O_2_ treatment was used as control in the apoptosis assay. The adherent cells were collected by trypsinization after 22 h. The collected cells were washed using PBS and then stained with 195 µl binding buffer (1×), 5 µl Annexin V-FITC, and 5 µl 7-AAD at room temperature for 10 min according to the instruction manual. The samples were analyzed using the NovoCyte flow cytometer (ACEA Bioscience, USA). Early apoptosis was designated as annexin positive/7-AAD negative, and late apoptosis and necrosis were designated as annexin positive/7-AAD positive. All experiments were done in triplicate, and the results (average values of the three experiments) were expressed as a percentage of the cells.

Cell cycle analysis was performed in the SKMEL-28 cell line. Initially, 2.5 × 10^6^ cells were cultured in 60-mm cell culture plates with DMEM supplemented with 10% FBS. On the next day, the cells were transfected with empty pGL3 or pGL3_*STAT3*_C or pGL3_*STAT3_*G vectors of the *STAT3* c.-1937C>G SNV using PEI watering system (Veritas, USA). GFP vector was used to monitor the transfection, and the cell was cultured in an atmosphere of 5% CO_2_ at 37°C for 24 h. After 24 h, cells were collected by trypsinization and washed by centrifugation in PBS at 300 g for 10 min 4°C before these were permeabilized in 1 ml cold 70% (v/v) ethanol (1 h, 4°C). Following washes with 0.25% Triton X-100 (PFT), the cells were stained in 300 µl of PFT with 5 µl 7-AAD Viability Stain Solution (Invitrogen, USA). A control tube was prepared without antibody labeling. The samples were analyzed on NovoCyte flow cytometer (ACEA Bioscience, USA). Individual separation of the cells was performed using the Cell Cycle Plot tool of the Novoexpress software, which is an analysis of the intensity of the 7-AAD marking and separation of the G1, S, and G2 cell cycle phases. All experiments were done in triplicate, and the results were expressed as the percentage of cells (average of three experiments) in a particular cycle phase.

### Statistical analyses

Hardy–Weinberg equilibrium (HWE) was tested using chi-square (*χ* ^2^) for the goodness-to-fit test. Haploview 4.2 software (www.broad.mit.edu/mpg/haploview) was used to verify if the markers were properly included in the haplotype analysis. Linkage disequilibrium was measured by the disequilibrium coefficient (*D*′), and significance was considered at *D*′ ≥0.80. Differences between groups were analyzed by *χ* ^2^ or Fisher’s exact test. Multivariate analysis using the logistic regression model served to obtain age, nevi, and sun exposure status-adjusted crude odds ratios with 95% confidence intervals (CIs) in comparisons involving patients and controls. Bonferroni correction for multiple comparisons was used to adjust the *p*-values obtained in clinicopathological aspect analyses. Considering continuous variables, data sets were probed for normality using Shapiro–Wilk’s test; if the data set assumed normal distribution, *t*-test and ANOVA were used for analysis, but if it did not assume a normal distribution, Mann–Whitney and Kruskal–Wallis tests were used to compare the groups.

Progression-free survival (PFS) was calculated from the date of surgery until the date of first recurrence, progression of disease, death from any cause, or last follow-up. Melanoma-specific survival (MSS) was calculated from the date of diagnosis until the date of death from the disease or last follow-up. PFS and MSS times were calculated using Kaplan–Meier probabilities, and differences between curves were analyzed by log-rank test. The prognostic impact of age, gender, tumor location, type of growth, Clark level, Breslow thickness, tumor stage, and genotypes of *JAK1*, *JAK2*, and *STAT3* SNVs in the survival of patients was evaluated using Cox proportional hazard ratio regression. In a second step, all variables with *p <*0.15 were included in a multivariate Cox regression.

For the statistical tests, significance was two-sided and achieved when *p*-values were <0.05. The intensities of the protein bands were quantitated using ImageJ software (National Institutes of Health, USA), and all tests were performed using SPSS 21.0 software (SPSS Incorporation, IL, USA).

## Results

### Characteristics of the study population

The clinicopathological aspects of the patients and controls enrolled in the study are presented in **Table 1**. The controls were younger than the patients (median age: 47 vs. 55 years; *p* < 0.0001), and the patients had more nevi (59.7 vs. 17.2%, *p* < 0.0001), referred more sun exposure (79.0 vs. 44.9%, *p* < 0.0001), sunburn episodes (53.2 vs. 28.8%, *p* < 0.0001), and chronic sun exposure (48.8 vs. 24.8%, *p* < 0.0001) than the controls. Differences in age, number of nevi, sun exposure, and sunburn of individuals of each group were corrected in comparisons of patients and controls by multivariate analysis using the logistic regression model.

**Table 1:** Distribution of 248 cutaneous melanoma patients and 274 controls stratified by clinicopathological aspects.

Characteristic	Patients, *N* (%)	Controls, *N* (%)	*p*-value
Age (years)			
≤55	125 (50.4)	226 (82.5)	<**0.0001**
>55	123 (49.6)	48 (17.5)	
Gender			
Male	128 (51.6)	140 (51.1)	0.90
Female	120 (48.4)	134 (48.9)	
Nevi^a^			
<20	94 (37.9)	214 (78.1)	<**0.0001**
≥20	148 (59.7)	47 (17.2)	
Phototype^a^			
I or II	157 (63.3)	162 (59.1)	0.07
III to VI	74 (29.8)	107 (39.1)	
Sun exposure^a^			
Yes	196 (79.0)	123 (44.9)	<**0.0001**
No	41 (16.5)	146 (53.3)	
Sunburn episodes^a^			
Yes	132 (53.2)	79 (28.8)	<**0.0001**
No	93 (37.5)	190 (69.3)	
Type of sun exposure^a^			
No/intermittent	97 (39.1)	201 (73.4)	<**0.0001**
Chronic	121 (48.8)	68 (24.8)	
Tumor location			
Limbs	81 (32.7)	NA	
Axial	167 (67.3)		
Ulceration^a^			
Yes	68 (27.4)	NA	
No	107 (43.2)		
Type of growth^a^			
Vertical	111 (44.8)	NA	
Horizontal	33 (13.3)		
Clark level^a^			
I or II	74 (29.8)	NA	
III to V	156 (63.0)		
Breslow thickness (mm)^a^			
≤1.5 mm	118 (47.6)	NA	
>1.5 mm	102 (41.1)		
Clinical stage^a,b^			
0 to II	164 (66.2)	NA	
III or IV	56 (22.6)		
Metastasis^a^			
Yes	18 (7.3)	NA	
No	183 (73.8)		

Significant values are presented in bold.

N, number of individuals; %, percentage; mm, millimeters; NA, not applicable.

a The number differed from the total quoted in the study because it was not possible to obtain consistent information in some cases.

b Clinical stage was classified by the American Joint Committee on Cancer criteria.

### Molecular characteristics of cell lines

The MMGP3, MMSK22, MMLN24, and UACC903 cell lines presented the CC genotype, the A375, SKMEL-103, MMLN9, and MMLN10 cell lines presented the CG genotype, and the G361, SKMEL-28, MSK8, MMLN14, MMLN23, and MEWO cell lines presented the GG genotype of the *STAT3* c.-1937C>G SNV.

Mutations of *NRAS* and *BRAF* genes were seen in G361, A375, SKMEL-103, and UACC903 cell lines, *BRAF* gene mutation was seen in SKMEL-28, and *RAS* gene mutation was seen in MMNL9 and MMNL10 cell lines; no mutations of *RAS* and *BRAF* were characterized in the MEWO cell line, and gene mutations were not analyzed in the remaining cell lines (**Supplementary Table S4**).

### SNVs and CM risk

The patients’ and controls’ samples were in HWE for the loci *JAK1* c.1648+1272G>A (*X* ^2^ = 2.44, *p* = 0.11; *X* ^2^ = 0.02, *p* = 0.88), *JAK1* c.991-27C>T (*X* ^2^ = 1.47, *p* = 0.22; *X* ^2^ = 1.69*, p* = 0.19), *JAK2* c.-1132G>T (*X* ^2^ = 0.79, *p* = 0.37; *X* ^2^ = 2.73, *p* = 0.09), *JAK2* c.-139G>A (*X* ^2^ = 0.01, *p*= 0.92; *X* ^2^ = 0.001, *p* = 0.97), *STAT3* c.*1671T>C (*X* ^2^ = 0.24, *p* = 0.62; *X* ^2^ = 1.59, *p* = 0.20), and *STAT3* c.-1937C>G (*X* ^2^ = 0.63*, p* = 0.42; *X* ^2^ = 0.004, *p* = 0.94), respectively.

The frequencies of *JAK1*, *JAK2*, and *STAT3* genotypes and respective alleles in patients and controls are presented in **Table 2**. Individuals with *STAT3* c*1671TT and *STAT3* c.-1937CC genotypes were under 1.70 and 1.60-fold increased risks for CM than those with the remaining genotypes.

**Table 2:** *JAK1*, *JAK2* and *STAT3* genotypes and alleles in 248 cutaneous melanoma patients and 274 controls.

Genotype/allele	Patients *N* (%)	Controls *N* (%)	*p*-value	OR^a^ (95% CI)
*JAK1* c.1648+1272G>A				
GG	99 (39.9)	100 (36.5)	0.29	Reference
GA or AA	149 (60.1)	174 (63.5)		1.28 (0.81–2.02)
GG or GA	205 (82.7)	230 (83.9)	0.41	Reference
AA	43 (17.3)	44 (16.1)		1.27 (0.70–2.28)
G allele	0.61	0.60	0.76	Reference
A allele	0.39	0.40		1.05 (0.76–1.44)
*JAK1* c.991-27C>T				
CC	122 (49.2)	118 (43.1)	0.28	Reference
CT or TT	126 (50.8)	156 (56.9)		1.27 (0.81–1.99)
CC or CT	220 (88.7)	234 (85.4)	0.94	Reference
TT	28 (11.3)	40 (14.6)		1.02 (0.53–1.95)
C allele	0.69	0.64	0.40	Reference
T allele	0.31	0.36		1.15 (0.82–1.60)
*JAK2* c.-1132G>T				
GG	109 (44.0)	127 (46.4)	0.33	Reference
GT or TT	139 (56.0)	147 (53.6)		1.24 (0.79–1.95)
GG or GT	215 (86.7)	237 (86.5)	0.78	Reference
TT	33 (13.3)	37 (13.5)		1.09 (0.56–2.11)
G allele	0.65	0.66	0.55	Reference
T allele	0.35	0.34		1.10 (0.79–1.54)
*JAK2* c.-139G>A				
GG	244 (98.4)	273 (99.6)	0.25	Reference
GA or AA	04 (1.6)	01 (0.4)		6.09 (0.26–139.20)
GG or GA	248 (100.0)	274 (100.0)	NC	Reference
AA	00 (0.0)	00 (0.0)		NC
G allele	0.99	1.00	0.25	Reference
A allele	0.01	0.00		6.04 (0.26–137.27)
*STAT3* c.*1671T>C				
TT	89 (35.9)	83 (30.3)	**0.02**	1.70 (1.05–2.75)
TC or CC	159 (64.1)	191 (69.7)		Reference
TT or TC	205 (82.7)	209 (76.3)	0.43	1.24 (0.71–2.15)
CC	43 (17.3)	65 (23.7)		Reference
T allele	0.59	0.53	0.05	1.36 (1.00–1.87)
C allele	0.41	0.47		Reference
*STAT3* c.-1937C>G				
CC	131 (52.8)	120 (43.8)	**0.03**	1.60 (1.02–2.51)
CG or GG	117 (47.2)	154 (56.2)		Reference
CC or CG	226 (91.1)	243 (88.7)	0.77	1.11 (0.54–2.28)
GG	22 (8.9)	31 (11.3)		Reference
C allele	0.72	0.66	0.15	1.28 (0.91–1.79)
G allele	0.28	0.34		Reference

Significant values are presented in bold.

N, number of cases; %, percentage; CI, confidence interval; NC, not calculated.

a Odds ratio (OR) was adjusted by age, nevi, and sun exposure by multivariate analysis.

The frequencies of combined genotypes and significant combined genotypes in patients and controls are shown in **Supplementary Table S5** and **Table 3**, respectively. In combinations of two SNVs, individuals with *JAK1* c.1648+1272GG plus *STAT3* c.*1671TT, *JAK1* c.1648+1272GG plus *STAT3* c.-1937CC, *JAK1* c.991-27CC plus *STAT3* c.*1671TT, *JAK1* c.991-27CC plus *STAT3* c.-1937CC, and *STAT3* c.*1671TT plus *STAT3* c.-1937CC genotypes had 2.54-, 2.15-, 2.32-, 2.10-, and 1.90-fold increased risks of developing CM than those with the remaining genotypes, respectively. Individuals with *JAK1* c.1648+1272GG plus *JAK1* c.991-27CC plus *STAT3* c.*1671TT and *JAK1* c.1648+1272GG plus *JAK1* c.991-27CC plus *STAT3* c.-1937CC genotypes were under 2.66- and 2.35-fold increased risks of CM development than others, respectively, when combinations of three SNVs were considered. In combinations of four SNVs, individuals with *JAK1* c.1648+1272GG plus *JAK1* c.991-27CC plus *JAK2* c.-1132GG plus *STAT3* c.*1671TT, *JAK1* c.1648+1272GG plus *JAK1* c.991-27CC plus *JAK2* c.-1132GG plus *STAT3* c.-1937CC, *JAK1* c.1648+1272GG plus *JAK1* c.991-27CC plus *STAT3* c.*1671TT plus *STAT3* c.-1937CC >G, and *JAK1* c.991-27CC plus *JAK2* c.-1132GG plus *STAT3* c.*1671TT plus *STAT3* c.-1937CC genotypes had 3.56-, 3.21-, 3.95-, and 3.67-fold increased risks of CM incidence than those with the remaining genotypes, respectively.

**Table 3:** *JAK1*, *JAK2*, and *STAT3* significant combined genotypes in 248 cutaneous melanoma patients and 274 controls.

Genotype	Patients *N* (%)	Controls *N* (%)	*p-*value	OR^a^ (95% CI)
*JAK1* c.1648+1272G>A + *STAT3*c.*1671T>C				
GG + TT	38 (27.9)	29 (19.5)	**0.01**	2.54 (1.23–5.25)
GA or AA + TC or CC	98 (72.1)	120 (80.5)		Reference
GG or GA + TT or TC	182 (88.3)	208 (86.7)	0.72	1.13 (0.55–2.32)
AA + CC	24 (11.7)	32 (13.3)		Reference
*JAK1* c.1648+1272G>A + *STAT3* c.-1937C>G				
GG + CC	55 (43.0)	44 (31.0)	**0.02**	2.15 (1.12–4.13)
GA or AA + CG or GG	73 (57.0)	98 (69.0)		Reference
GG or GA + CC or CG	188 (97.4)	204 (97.6)	0.12	3.45 (0.71–16.63)
AA + GG	05 (2.6)	05 (2.4)		Reference
*JAK1* c.991-27C>T + *STAT3* c.*1671T>C				
CC + TT	46 (35.7)	34 (24.1)	**0.01**	2.32 (1.16–4.64)
CT or TT + TC or CC	83 (64.3)	107 (75.9)		Reference
CC or CT + TT or TC	183 (96.8)	185 (92.0)	0.46	1.49 (0.51–4.35)
TT + CC	06 (3.2)	16 (8.0)		Reference
*JAK1* c.991-27C>T + *STAT3* c.-1937C>G				
CC + CC	67 (51.9)	52 (37.1)	**0.02**	2.10 (1.10–4.00)
CT or TT + CG or GG	62 (48.1)	88 (62.9)		Reference
CC or CT + CC or CG	200 (99.0)	208 (97.7)	0.48	1.97 (0.28–13.68)
TT + GG	02 (1.0)	05 (2.3)		Reference
*STAT3* c.*1671T>C + *STAT3* c.-1937C>G				
TT + CC	82 (42.7)	74 (33.8)	**0.01**	1.90 (1.13–3.19)
TC or CC + CG or GG	110 (57.3)	145 (66.2)		Reference
TT or TC + CC or CG	201 (91.8)	202 (89.4)	0.70	1.17 (0.51–2.65)
CC + GG	18 (8.2)	24 (10.6)		Reference
*JAK1* c.1648+1272G>A + *JAK1* c.991-27C>T + *STAT3* c.*1671T>C				
GG + CC + TT	38 (31.4)	29 (21.3)	**0.01**	2.66 (1.25–5.62)
GA or AA + CT or TT +TC or CC	83 (68.6)	107 (78.7)		Reference
GG or GA + CC or CT + TT or TC	172 (96.6)	183 (92.0)	0.51	1.42 (0.49–4.17)
AA + TT + CC	06 (3.4)	16 (8.0)		Reference
*JAK1* c.1648+1272G>A + *JAK1* c.991-27C>T + *STAT3* c.-1937C>G				
GG + CC + CC	55 (47.0)	44 (33.3)	**0.01**	2.35 (1.18–4.67)
GA or AA + CT or TT + CG or GG	62 (53.0)	88 (66.7)		Reference
GG or GA + CC or CT + CC or CG	188 (98.9)	204 (97.6)	0.47	2.02 (0.29–14.12)
AA + TT + GG	02 (1.1)	05 (2.4)		
*JAK1* c.1648+1272G>A + *JAK1* c.991-27C>T + *JAK2* c.-1132G>T + *STAT3* c.*1671T>C				
GG + CC + GG + TT	19 (28.8)	11 (14.9)	**0.02**	3.56 (1.22–10.37)
GA or AA + CT or TT + GT or TT + TC or CC	47 (71.2)	63 (85.15)		Reference
*JAK1* c.1648+1272G>A + *JAK1* c.991-27C>T + *JAK2* c.-1132G>T + *STAT3* c.-1937C>G				
GG + CC + GG + CC	28 (45.9)	21 (29.2)	**0.01**	3.21 (1.22–8.47)
GA or AA + CT or TT + GT or TT + CG or GG	33 (54.1)	51 (70.8)		Reference
*JAK1* c.1648+1272G>A + *JAK1* c.991-27C>T + *STAT3* c.*1671T>C + *STAT3* c.-1937C>G				
GG + CC + TT + CC	34 (36.6)	24 (22.2)	**0.002**	3.95 (1.66–8.95)
GA or AA +CT or TT + TC or CC + CG or GG	59 (63.4)	84 (77.8)		Reference
*JAK1* c.991-27C>T + *JAK2* c.-1132G>T + *STAT3* c.*1671T>C + *STAT3* c.-1937C>G				
CC + GG + TT + CC	20 (38.5)	12 (19.0)	**0.01**	3.67 (1.25–10.79)
CT or TT + GT or TT + TC or CC + CG or GG	32 (61.5)	51 (81.0)		Reference

Significant values are presented in bold.

N, number of cases; %, percentage; CI, confidence interval.

a Odds ratio (OR) adjusted by age, nevi, and sun exposure by multivariate analysis.

Individuals with TC haplotype of *STAT3* c*1671T>C and *STAT3* c.-1937C>G SNVs were under 1.64-fold increased risk for CM than those with the remaining haplotypes of *STAT3* SNVs (**Table 4**).

**Table 4:** *JAK1*, *JAK2*, and *STAT3* haplotypes in 248 cutaneous melanoma patients and 274 controls.

Haplotype		Patients’ frequency	Controls’ frequency	*p-*value	OR^a^ (95% CI)
*JAK1*c.1648+1272G>A	*JAK1* c.991-27C>T				
G	C	0.61	0.60	0.76	1.05 (0.76–1.44)
G	T	0.99	1.00	1.00	NC
A	C	0.07	0.04	0.30	1.40 (0.73–2.70)
A	T	0.31	0.36	0.40	1.15 (0.82–1.60)
*JAK2* c.-1132G>T	*JAK2* c.-139G>A				
G	G	0.65	0.66	0.52	1.11 (0.79–1.55)
G	A	0.002	0.001	0.55	3.28 (0.06–171.59)
T	G	0.34	0.34	0.63	1.08 (0.77–1.51)
T	A	0.006	0.00	0.99	NC
*STAT3* c.*1671T>C	*STAT3* c.-1937C>G				
T	C	0.43	0.35	**0.003**	1.64 (1.19–2.27)
T	G	0.16	0.18	0.16	1.35 (0.88–2.06)
C	C	0.29	0.31	0.07	1.36 (0.96–1.93)
C	G	0.12	0.16	0.67	1.09 (0.70–1.71)

Significant values are presented in bold.

%, percentage; CI, confidence interval; NC, not calculated.

a Odds ratio (OR) adjusted by age, nevi, and sun exposure by multivariate analysis.

### SNVs and clinicopathological aspects of patients

*JAK1* c.991-27CC genotype was more common in male than in female patients (57.8 vs. 40.0%, *p* = 0.005). The frequency of CC genotype was also higher in male patients than in controls (57.8 vs. 37.1%); individuals with CC genotype were under 2.12-fold increased risk for CM than individuals with other genotypes (95% CI: 1.14–3.94,*p* = 0.01). Similar frequencies of *JAK1*, *JAK2*, and *STAT3* genotypes were seen in patients stratified by age, phototype, nevi presence, sun exposure, sunburn episodes, type of sun exposure (**Supplementary Table S6**), tumor location, ulceration, type of growth, Clark level, Breslow thickness, clinical stage, and metastases (**Supplementary Table S7**). No associations of *JAK1*, *JAK2*, and *STAT3* combined genotypes, alleles, and haplotypes with the clinicopathological aspects of patients were also seen in the study (data not shown).

### Survival analysis

Survival data were obtained from 237 out of 248 CM patients. The median follow-up of patients enrolled in the survival analysis was 101 months (range, 5–249 months). The final status of patients was established in July 2021. At that time, 148 patients were alive without disease, two were alive with disease, 52 died due to CM effects, and 35 died due to unrelated causes.

At 60 months of follow-up, PFS was lower in male patients (61.0 vs. 75.0%, *p* = 0.008), in patients with axial tumor (trunk and head) (63.5 vs. 76.9%, *p* = 0.05), vertical growth tumor (66.7 vs. 87.9%, *p* = 0.01), Clark levels III to V tumors (59.7 vs. 90.3%, *p* < 0.0001), Breslow thickness higher than 1.5 mm (46.4 vs. 89.4%, *p* < 0.0001), and advanced-stage tumors (stage III or IV) (25.6 vs. 79.4%, *p* < 0.0001) (Kaplan Meier estimates) (**Supplementary Figure S2**). Differences among groups remained the same in the univariate Cox analysis. After multivariate Cox analysis, patients with axial tumor, with Breslow thickness higher than 1.5 mm, and advanced stages had 2.42, 4.26, and 4.08 more chances of presenting disease progression, respectively (**Supplementary Table S8**).

At 60 months of follow-up, MSS was lower in older patients (78.3 vs. 88.0%, *p* = 0.02), male patients (74.0 vs. 92.8%, *p* < 0.0001), patients with axial tumor (79.2 vs. 91.8%, *p* = 0.005), Clark levels III to V tumors (78.6 vs. 97.2%, *p* < 0.0001), Breslow thickness higher than 1.5 mm (69.1 vs. 99.1%, *p* < 0.0001), and advanced-stage tumors (50.3 vs. 92.5%, *p* < 0.0001) (Kaplan–Meier estimates) (**Supplementary Figure S3**). Differences among groups remained the same in the univariate Cox analysis. After the multivariate Cox analysis, patients with axial tumor, Breslow thickness higher than 1.5 mm, and advanced stages had 4.52, 7.66, and 3.95 more chances of evolving to death due to CM than others, respectively (**Supplementary Table S8**).

*JAK1* (c.1648+1272G>A, c.991-27C>T), *JAK2* (c.-1132G>T, c.-139G>A) and *STAT3* (c.*1671T>C, c.-1937C>G) did not alter the PFS and MSS of CM patients (**Supplementary Table S8**). No association of SNV combinations, gene alleles, and haplotypes with PFS or MSS was found in the study (data not shown).

### *JAK1*, *JAK2*, and *STAT3* expression in peripheral blood samples

**Figure 1:**
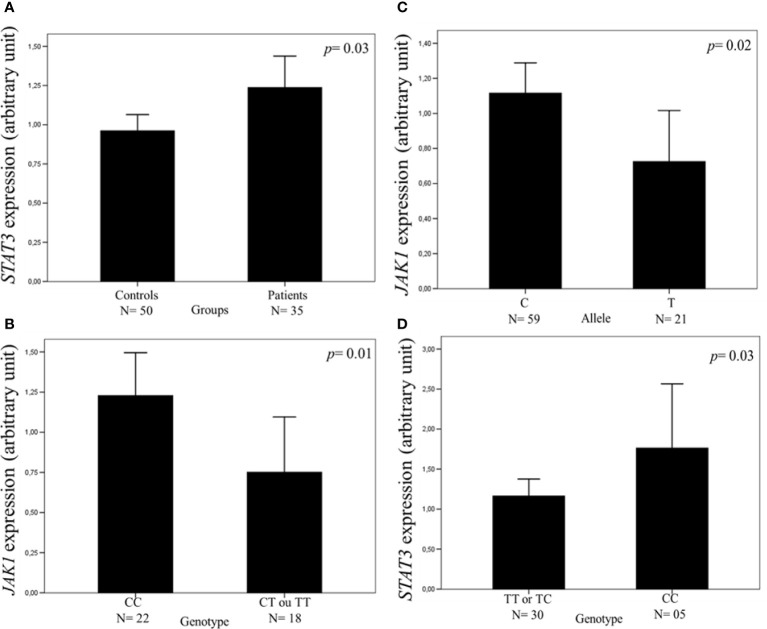
Expression of the *JAK1* and *STAT3* genes in the leukocytes of peripheral blood samples measured by real-time polymerase chain reaction. The relative expression level was normalized by β-actin reference gene with the 2^-DDCt^ cycle threshold method. The values of 15% of the samples were repeated in separate experiments with 100% agreement, and the results are expressed in arbitrary units (AUs). The results are shown as means between groups. For the statistical tests, values with *p <*0.05 were considered significant. The *STAT3* expression was higher in patients with cutaneous melanoma (CM) than in controls (1.23 vs. 0.96 AU) **(A)**. The *JAK1* expression was higher in CM patients with CC genotype **(B)** and with allele C **(C)** than in those with CT or TT genotype and allele T of *JAK1* c.991-27C>T, the single-nucleotide variant (SNV) (1.22 vs. 0.75 and 1.11 vs. 0.75 AU, respectively). The *STAT3* expression was higher in CM patients with CC genotype than in those with TT or TC genotype of c.*1671T>C SNV (1.76 vs. 1.16 AU) **(D)**.

Similar mean values of *JAK1* (1.02 vs. 1.26 AU, *p* = 0.08) and *JAK2* (1.00 vs. 1.19 AU, *p* = 0.32) expressions were found in the leukocytes of peripheral blood samples of patients and controls, but the mean value of *STAT3* expression was higher in patients than in controls (1.23 vs. 0.96 AU, *p* = 0.03) (**Figure 1A**).

The mean value of *JAK1* expression was higher in patients with the CC genotype (1.22 vs. 0.75 AU, *p* = 0.01) and C allele (1.11 vs. 0.75 AU, *p* = 0.02) of *JAK1* c.991-27C>T SNV than in those with the CT or TT genotype and T allele, respectively (**Figures 1B, C**). The mean value of *STAT3* expression was higher in patients with *STAT3* c.*1671CC genotype than in those with TT or TC genotype (1.76 vs. 1.16 AU, *p* = 0.03) (**Figure 1D**). Similar mean values of gene expression were seen in the leukocytes of the peripheral blood samples of CM patients with the distinct genotypes of *JAK1* c.1648+1272G>A, *JAK2* c.-1132G>T, *JAK2* c.-139G>A, and *STAT3* c.-1937C>G SNVs (**Supplementary Figure S4**, **Supplementary Table S9**).

From the results found in the associations of analyzed SNVs with risk of CM, *JAK1* c.991-27C>T and *STAT3* c.-1937C>G were seen in most of the significant combined genotypes. *STAT3* c.-1937C>G was considered as the SNV of greatest interest in the study; therefore, it was the object of additional functional analyzes.

### *STAT3* promoter region activity and expression in modified SKMEL-28 cell line

**Figure 2:**
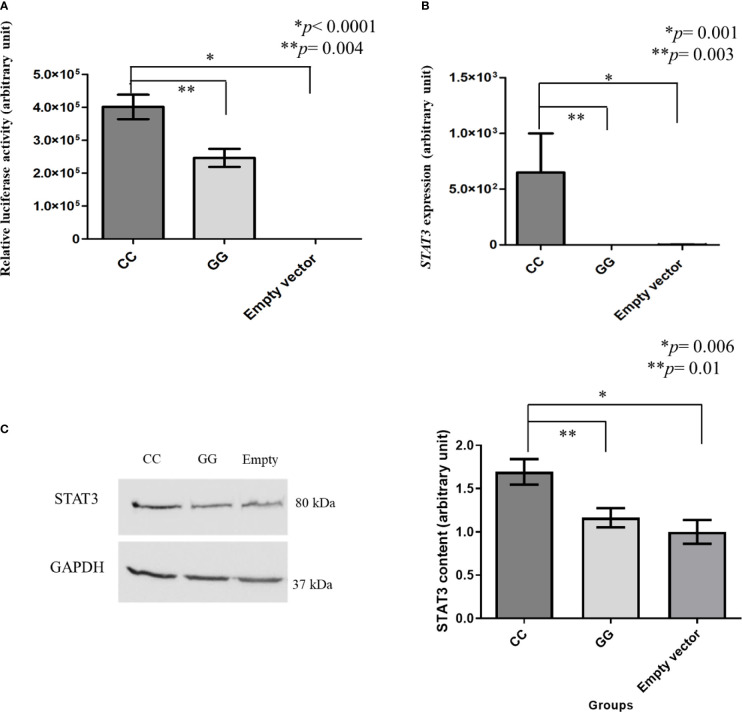
Luciferase activity and *STAT3* expression measured by real-time polymerase chain reaction and STAT3 level measured by western blot in modified SKMEL-28 (*STAT3* c.-1937CC or GG genotype). Total proteins (50 µg) were analyzed by 10% sodium dodecyl sulfate polyacrylamide gel electrophoresis, and the separated proteins were transferred to nitrocellulose membranes. The membranes were blocked with 5% skimmed milk in phosphate-buffered saline (PBS-Tween) and incubated with specific primary antibodies anti-STAT3 (Santa Cruz, USA) and anti-GAPDH (Santa Cruz, USA) overnight at 4°C. A horseradish peroxidase-conjugated goat anti-mouse IgG was used as the secondary antibody (Santa Cruz, USA). GAPDH was used as normalizer in the western blot experiments. ECL Western Blot Detection Reagents kit (GE Healthcare, USA) was used, and the intensities of the protein bands were quantitated using ImageJ software (National Institutes of Health, USA). The results are shown as means between groups. For the statistical tests, values with *p < *0.05 were considered significant. SKMEL-28 cells with CC genotype presented a higher *STAT3* luciferase activity than those with GG genotype [4,013.34 *vs.* 2,463.32 arbitrary units (AU)] and the one with empty vector (4,013.34 vs. 0.0122 AU) **(A)**, a higher *STAT3* gene expression than those with GG genotype (649.20 vs. 0.03 AU) and the one with empty vector (649.20 vs. 2.11 AU) **(B)**, and a higher STAT3 protein level than in those with GG genotype (1.69 *versus* 1.16 AU) and the one with empty vector (1.69 vs. 1.00 AU) **(C)**. * and ** statistically significant result.

The mean value of luciferase promoter region activity in SKMEL-28 cells with *STAT3* c.-1937CC genotype was higher than that found in cells with *STAT3* c.-1937GG genotype (4,013.34 *vs.* 2,463.32 UA; *p* = 0.004) (**Figure 2A**).

The mean value of *STAT3* expression in SKMEL-28 cells with *STAT3* c.-1937CC genotype was higher than those found in cells with GG genotype (649.20 vs. 0.03 AU; *p* = 0.003) and in cells with empty vector (649.20 vs. 2.11 AU; *p* = 0.001) (**Figure 2B**).

### STAT3 protein level in modified SKMEL-28 and unmodified cell lines

The STAT3 level was higher in SKMEL-28 cells with *STAT3* c.-1937CC genotype than in those with GG genotype (1.69 *versus* 1.16 AU; *p* = 0.01) and in those with empty vector (1.69 *vs*. 1.00 AU; *p* = 0.006) (**Figure 2C**).

Unmodified cell lines with *STAT3* c.-1937CC genotype also presented a higher STAT3 level than those with GG genotype (1.93 *versus* 1.27 UA, *p* = 0.0027) (**Supplementary Figure S5**) despite the status of *RAS* and *BRAF* mutations.

### STAT3 in apoptosis and cell cycle assays in modified SKMEL-28 cell line

**Figure 3:**
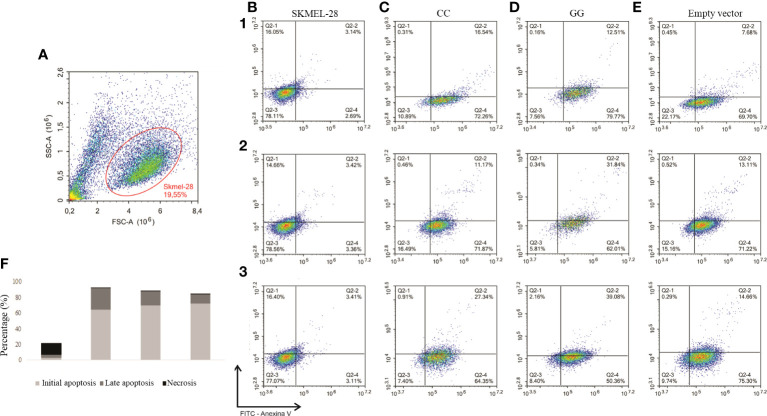
Detection of early and late apoptosis by flow cytometry in SKMEL-28 cutaneous melanoma cell line genetically modified to present the different genotypes of *STAT3* c.-1937C> G. The cells were subjected to nutrient deprivation, treated with 200 µM of hydrogen peroxide for 24 h, and marked with the antibodies Annexin V and 7-AAD. Three independent experiments were performed (1, 2, and 3). The identification of cells was performed by separation by size (FSC) and granularity (SSC) **(A)**. Dot plots referring to experiments one to three for the analysis of cell apoptosis, in which viable cells (Q2-3), cells in initial apoptosis (Q2-4), cells in late apoptosis (Q2-2), and necrosis (Q2) were identified in SKMEL-28 **(B)**, cell with CC genotype **(C)**, cell with GG genotype **(D)**, and cell transfection with empty vector **(E)**. For the statistical tests, values with *p <*0.05 were considered significant. The results are shown as means between groups: CC vs. GG (initial apoptosis: 69.49 vs. 64.05%, *p* = 0.57; late apoptosis: 18.35 vs. 27.81%, *p* = 0.36; necrosis: 0.56 vs. 0.89%, *p* = 0.64), CC vs. empty vector (initial apoptosis: 69.49 vs. 72.07%, *p* = 0.44; late apoptosis: 18.35 vs. 11.82%, *p* = 0.27; necrosis 0.56 vs. 0.42%, *p* = 0.50), and GG vs. empty vector (initial apoptosis: 64.05 vs. 72.07%, *p* = 0.40; late apoptosis: 27.81 vs. 11.82%, *p* = 0.12; necrosis 0.89 vs. 0.42%, *p* = 0.54) **(F)**.

Initially, cells were identified by size (forward scatter) and granularity (side scatter) as shown in **Figure 3A**; then, they were evaluated for specific tags. The percentages of live cells (Q2-3) in initial apoptosis (Q2-4), in late apoptosis (Q2-2), and in necrosis (Q2-1) are shown in **Figures 3B–E**. Similar percentages of apoptotic and necrotic cells were seen in SKMEL-28 with CC and GG genotypes of *STAT3* c.-1937C>G SNV within a 24-h period (**Figure 3F**).

**Figure 4:**
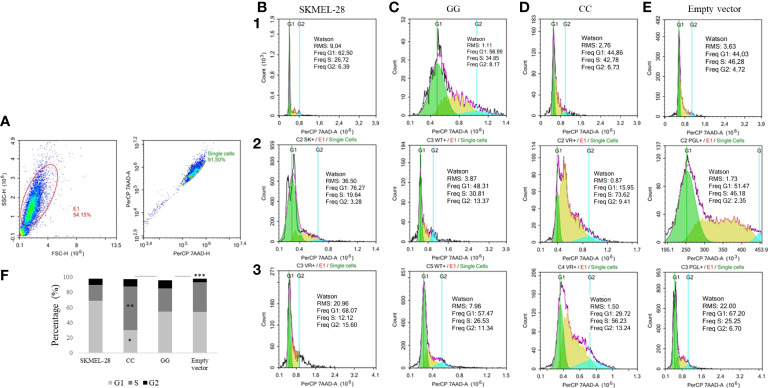
Cell cycle detection by flow cytometry in a SKMEL-28 cutaneous melanoma cell genetically modified to present the different genotypes of *STAT3* c.-1937C>G. The cells were stained with the 7-AAD antibody for cell viability. Three independent experiments were performed (1, 2, and 3). The identification of the cells was carried out from the separation by size (FSC) and granularity (SSC). Afterwards, the removal of the doublets was carried out through the area (A) and the height (H) **(A)**. From the individualized cells, the fluorescence intensity of 7-AAD and the separation of phases G1, S, and G2 of the cell cycle of experiments one to three were evaluated using the Watson model in SKMEL-28 **(B)**, cell with GG genotype **(C)**, cell with CC genotype **(D)**, and cell transfection with empty vector **(E)**. For the statistical tests, values with *p <*0.05 were considered significant. The results are shown as means between groups: CC vs. GG (G1: 30.18 vs. 54.26%, *p = 0.05; S: 57.54 vs. 30.73%, **p = 0.04; G2: 9.79 vs. 10.96%, *p* = 0.65), CC *vs*. empty vector (G1: 30.18 vs. 54.23%, *p* = 0.09; S: 57.54 vs. 39.24%, *p* = 0.18; G2: 9.79 vs. 4.59%, *p* = 0.08), and GG vs. empty vector (G1: 54.26 vs. 54.23%, *p* = 0.99; S: 30.73 vs. 39.24%, *p* = 0.31; G2: 10.96 vs. 4.59%, ***p = 0.03) **(F)**.

The cell cycle characteristics of SKMEL-28 line were quantified and evaluated using area (*A*) and height (*H*) for 7-AAD tagging, as shown in **Figure 4A**, and separation of the G1, S, and G2 phases of the cell cycle is shown in **Figures 4B–E**. The percentage of cells in S phase was higher than in SKMEL-28 cells with CC genotype than in SKMEL-28 cells with GG genotype of *STAT3* c.-1937C>G SNV (57.54 vs. 30.73%, *p* = 0.04). The percentage of cells in G2 phase was higher in SKMEL-28 cells with *STAT3* c.-1937GG genotype than in cells transfected with the empty vector (10.96 *versus* 4.59%, *p* = 0.03) (**Figure 4F**).

## Discussion

Our group investigated, in the current study, whether *JAK1* (c.1648+1272G>A, c.991-27C>T), *JAK2* (c.-1132G>T, c.-139G>A), and *STAT3* (c.*1671T>C, c.-1937C>G) SNVs alter the risk, clinicopathological aspects, and prognosis of CM as well as the activity of respective proteins.

Firstly, we observed that individuals with TT genotype of *STAT3* c.*1671T>C SNV were under 1.60-fold increased risk for CM than individuals with other genotypes. Previous studies demonstrated that CC genotype conferred protection against gastric cancer (12), pancreatic cancer (13), tongue squamous cell carcinoma (14), and hepatocellular carcinoma in women (34). Allele C also protected against tongue squamous cell carcinoma (14). In contrast, the SNV did not alter the risk of non-small cell lung cancer (35), hepatocellular carcinoma (36), and cancer in general (37). The differences between our data and the results obtained in previous studies may be attributed to the heterogeneity of the evaluated tumors and their biological and ethnical differences.

In this study, *STAT3* expression in the leukocytes of peripheral blood samples was higher in patients with *STAT3* c.*1671CC genotype compared to patients with TT or CT genotype; this finding was not expected by us since STAT3 acts on cell proliferation and survival (2), and TT genotype was associated with an increased risk of CM. Lai et al.(14) found a higher *STAT3* gene and microRNA expression in tongue squamous cell carcinoma than in normal tissue; the authors also observed increased STAT3 levels in the tongue squamous cell carcinoma of patients with *STAT3* c.*1671TT genotype than in the ones with other genotypes (14). In addition, an increased expression of *STAT3* gene was seen in lymphoblastoid B cells with TT genotype than in cells with other genotypes (12). We are aware that the *STAT3* expression was evaluated only in peripheral blood samples in this study, and thus further evaluation of gene expression should be conducted in CM fragments or cells of patients with different *STAT3* c.*1671T>C genotypes to obtain consistent conclusions about the role of *STAT3* c.*1671T>C SNV on protein production.

Secondly, we found that individuals with CC genotype of the *STAT3* c.-1937C>G SNV were under 6.70-fold increased risk of CM than individuals with other genotypes. Previous studies demonstrated that GG genotype conferred protection against non-small cell lung cancer (35) and breast cancer (38) development. Other studies showed that carriers of GG genotype were under an increased risk of lung cancer (39) and basal cell carcinoma (40). The allele G was associated with an increased cervical cancer risk (41), and the risks of gastric cancer (12), hepatocellular carcinoma (34), and cancer in general (39, 42) were not altered by the *STAT3* c.-1937C>G SNV. The contrast in the results of the studies may be attributed again to tumor heterogeneity and the populations’ ethnical differences.

*STAT3* c.-1937C>G SNV is in the 5′ region of the *STAT3* gene, at position 1,633 base pairs ahead of the ATG site (15) and has been associated with increased transcription factor NKX2-5 in the binding site (15) and with unclear functional consequences in the encoded protein. We observed that *STAT3* expression was higher in CM patients than in controls. In SKMEL-28 cells genetically modified to express different genotypes of the *STAT3* c.-1937C>G SNV, we also observed that cells with CC genotype presented increased luciferase activity, *STAT3* gene expression, and STAT3 levels when compared to cells with the GG genotype. Different CM cell lines with CC genotype also showed higher STAT3 levels than the one with GG genotype in our study despite the differences in *RAS* and *BRAF* mutation status. Ito et al. (16) did not find differences in luciferase activity in metastatic renal carcinoma cells with distinct alleles of *STAT3* c.-1937C>G SNV, and no difference in *STAT3* expression was found in melanoma cells with distinct *STAT3* c.-1937C>G genotypes by Schrama et al. (15). It is possible that the association of the CC genotype of *STAT3* c.-1937C>G SNV with a higher STAT3 expression and levels in different CM cells and experiments in the current study makes our findings more reliable than those described by Schrama et al. Thus, we can infer that the increased risk of CM in patients with the CC genotype of *STAT3* c.-1937C>G SNV is attributed to greater STAT3 protein production.

We also observed that cells with CC genotype presented a higher percentage of cells in the S phase of the cell cycle when compared to cells with the GG genotype in modified SKMELL-28, but similar percentages of apoptotic and necrotic cells were seen in cells with CC and GG genotypes. To the best of our knowledge, there are no studies on the role of *STAT3* c.-1937C>G SNV in apoptosis and cell cycle in CM, and thus it is not possible to compare our results with the others previously described. However, it is already well known that the transition from the G1 phase of the cell cycle to the S phase is crucial for the control of cell proliferation, and its misregulation promotes oncogenesis (43, 44). Since the S phase is responsible for DNA replication, we believe that *STAT3* c.-1937CC genotype possibly increases the JAK/STAT pathway activity, with a greater chance of CM formation and progression, as seen in individuals with *STAT3* c.-1937CC genotype enrolled in our study. It is essential to comment that additional functional studies are required to confirm these hypotheses.

Thirdly, the TC haplotype of *STAT3* c.*1671T>C and *STAT3* c.-1937C>G SNVs was more common in patients than in controls, thus being considered a risk factor for CM. We also observed a progressive increase in the risk of CM (up to 4.0-fold) when combined SNVs were analyzed two by two, three by three, and four by four, demonstrating the importance of evaluating genetic changes together in the signaling pathway instead of them isolated. While the functional impact of a single SNV may be low, the interaction of several SNVs with a slightly increased or reduced functional activity eventually affects cancer risk (45). It is worth commenting that *STAT3* c.-1937C>G and *JAK1* c.991-27C>T SNVs were present in most of all significant genotypic combinations. Once that a high expression of JAK1 has been identified in individuals with *JAK1* c.991-27CC genotype in this study, we believe that *JAK1* c.991-27C>T SNV may have contributed to the STAT3 SNV in maintaining JAK/STAT pathway activation, presenting a greater chance of CM development and progression as a consequence.

Fourthly, we observed that male patients with *JAK1* c.991-27CC genotype were more common than in female patients and that men with this genotype were under 2.12-fold increased risk for CM than the controls. To the best of our knowledge, there are no studies of these SNVs associated with CM risk and clinicopathological aspects.

Finally, we found that age, gender, tumor location, pattern of tumor growth, Clark levels, Breslow thickness, and tumor stage altered the survival of our CM patients as previously reported (46–48). Nevertheless, patients’ survival was not altered by the analyzed SNVs. We are again aware that the number of patients with distinct genotypes of SNVs presenting disease progression and/or death may have been insufficient to evidence impacts of the inherited abnormalities in survival.

In conclusion, our data present, for the first time, preliminary evidence that *JAK1* (c.1648+1272G>A, c.991-27C>T), *JAK2* (c.-1132G>T), and *STAT3* (c.*1671T>C, c.-1937C>G) SNVs alter the risk and clinical aspect of CM patients, where *STAT3*c.-1937C>G *JAK1* c.991-27C>T has the most important action. The association of *STAT3* c.-1937C>G SNV with the risk of CM may be attributed to its actions in the promoter region of *STAT3* gene and in the cell cycle, with consequent changes in protein production and cell proliferation. We are aware that the results of the association of genotypes with the clinicopathological aspects and survival of CM patients were obtained from a relatively small number of patients from a single country, a single genetically transformed cell line served for the majority of functional analyses of *STAT3* c.-1937C>G SNV, the mechanisms by which the *STAT3* c.-1937C>G SNV operates in the cell cycle were not totally determined, and functional studies for other SNVs of interest were not performed in the current study. Thus, these results should be validated in a further epidemiological study with CM patients and controls of diverse ethnic populations and clarified in additional functional studies, and if they are, the data can be used to select individuals at a high risk of CM who should receive special attention in tumor prevention and early detection.

## Data availability statement

The datasets presented in this study can be found in online repositories. The names of the repository/repositories and accession number(s) can be found in the article/**Supplementary Material**.

## Ethics statement

The studies involving human participants were reviewed and approved by UNICAMP Research Ethics Committee (Process 58186316.1.0000.5404). The patients/participants provided their written informed consent to participate in this study.

## Author contributions

GG, RS, JR, and CL were responsible for the study design. GG, LM, KF, LC, JC, and AM were responsible for collecting clinical data and performing the experiment. GG, GL, LM, KF, CT, LC, and AM were responsible for the statistical analysis and interpretation of data. GG was responsible for drafting the manuscript and GL, RS, JR, and CL were responsible for the critical revision of the manuscript for intellectual content. All authors contributed to the article and approved the submitted version.

## Funding

This work was supported by grants from the FAPESP (2016/25407-4 and 2019/16776-4) and FAPESP 2019/09168-8.

## Conflict of interest

The authors declare that the research was conducted in the absence of any commercial or financial relationships that could be construed as a potential conflict of interest.

## Publisher’s note

All claims expressed in this article are solely those of the authors and do not necessarily represent those of their affiliated organizations, or those of the publisher, the editors and the reviewers. Any product that may be evaluated in this article, or claim that may be made by its manufacturer, is not guaranteed or endorsed by the publisher.
