# Supplementary material for: Association of JAK/STAT genetic variants with cutaneous melanoma
Source: Front Oncol. 2022 Aug 2;12:943483. doi: 10.3389/fonc.2022.943483 (PMC9379289; doi:10.3389/fonc.2022.943483)
Supplement: Supplementary file 1 [file DataSheet_1.pdf]

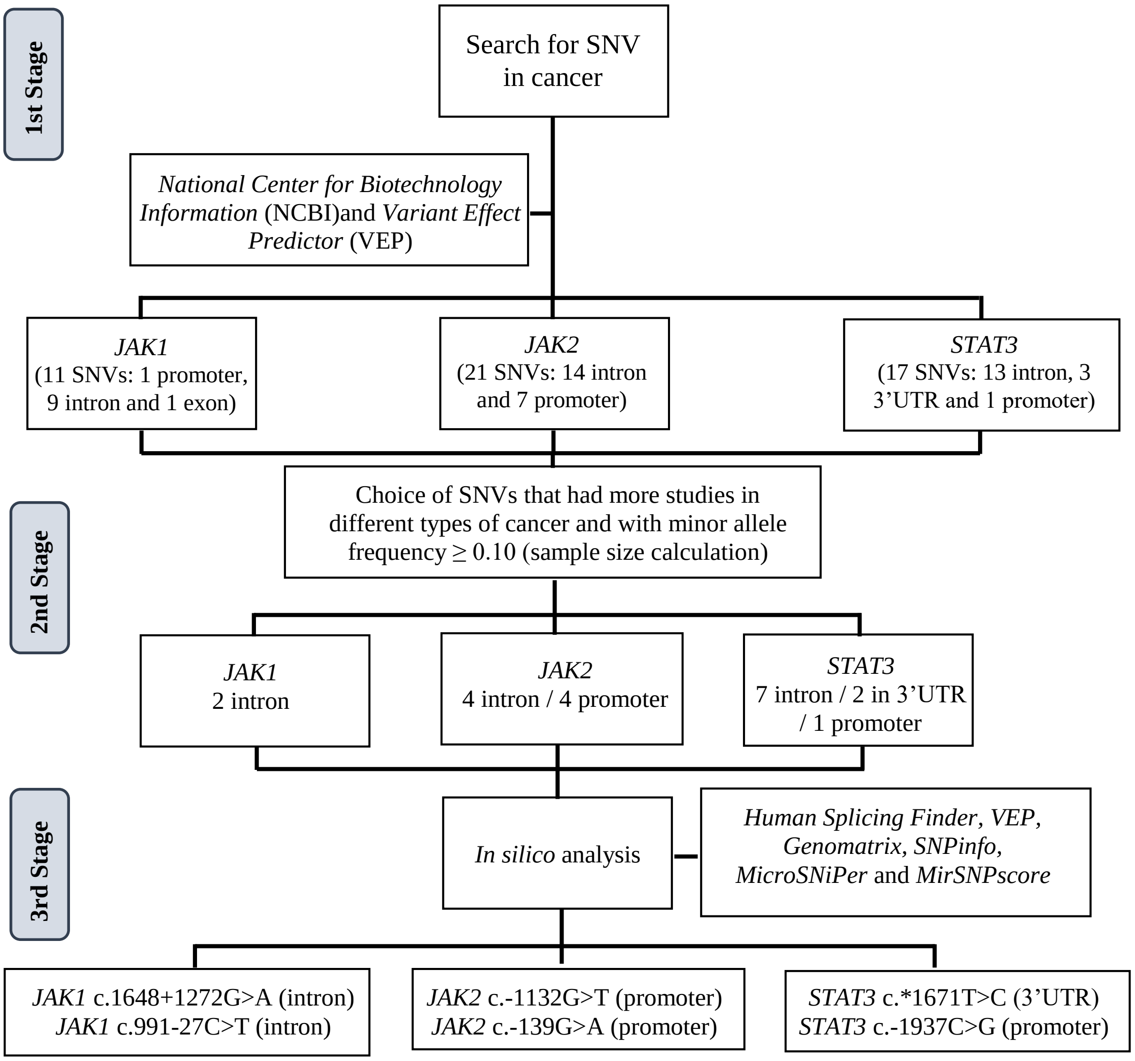

Supplementary Figure S1.

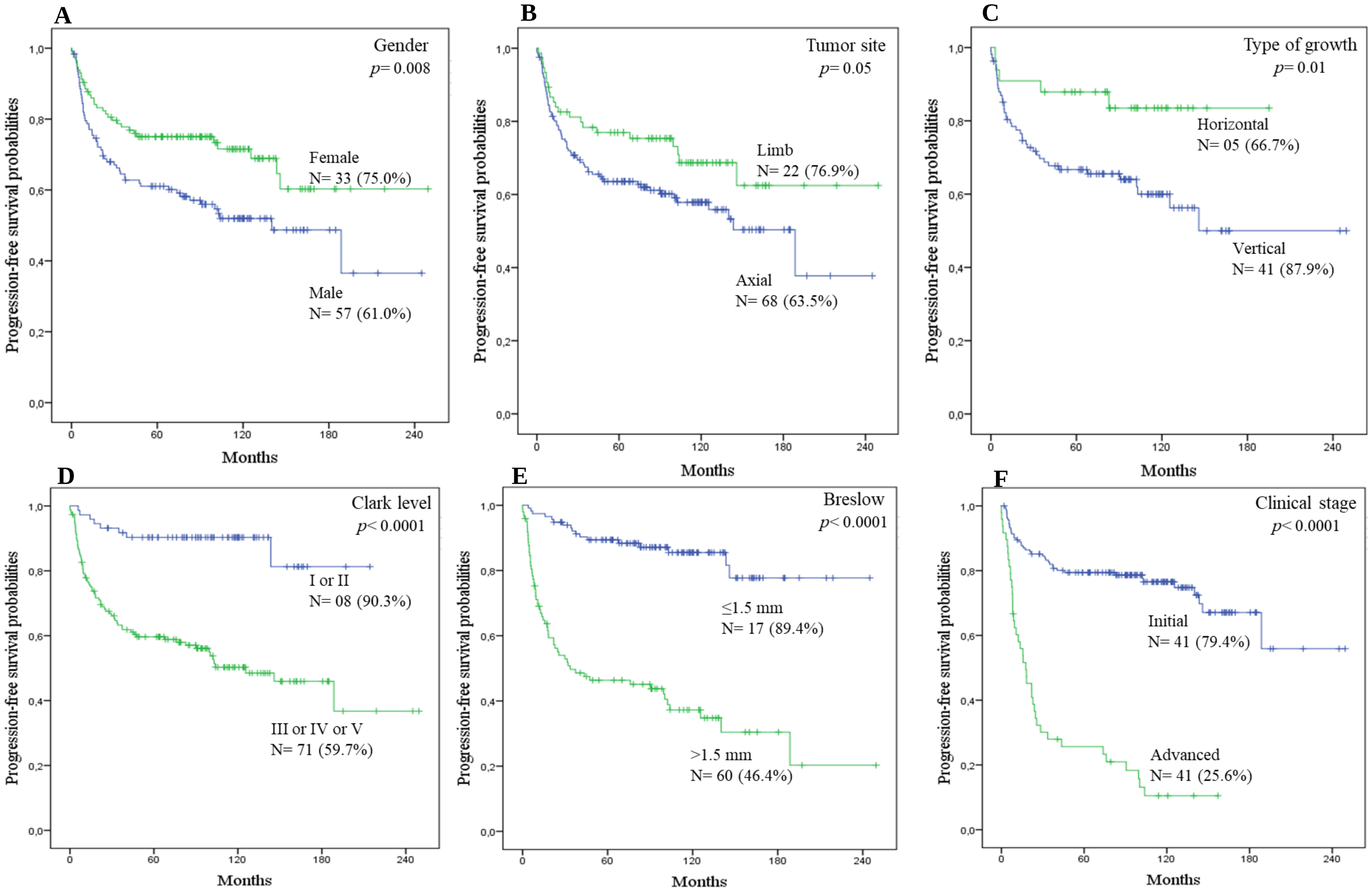

Supplementary Figure S2

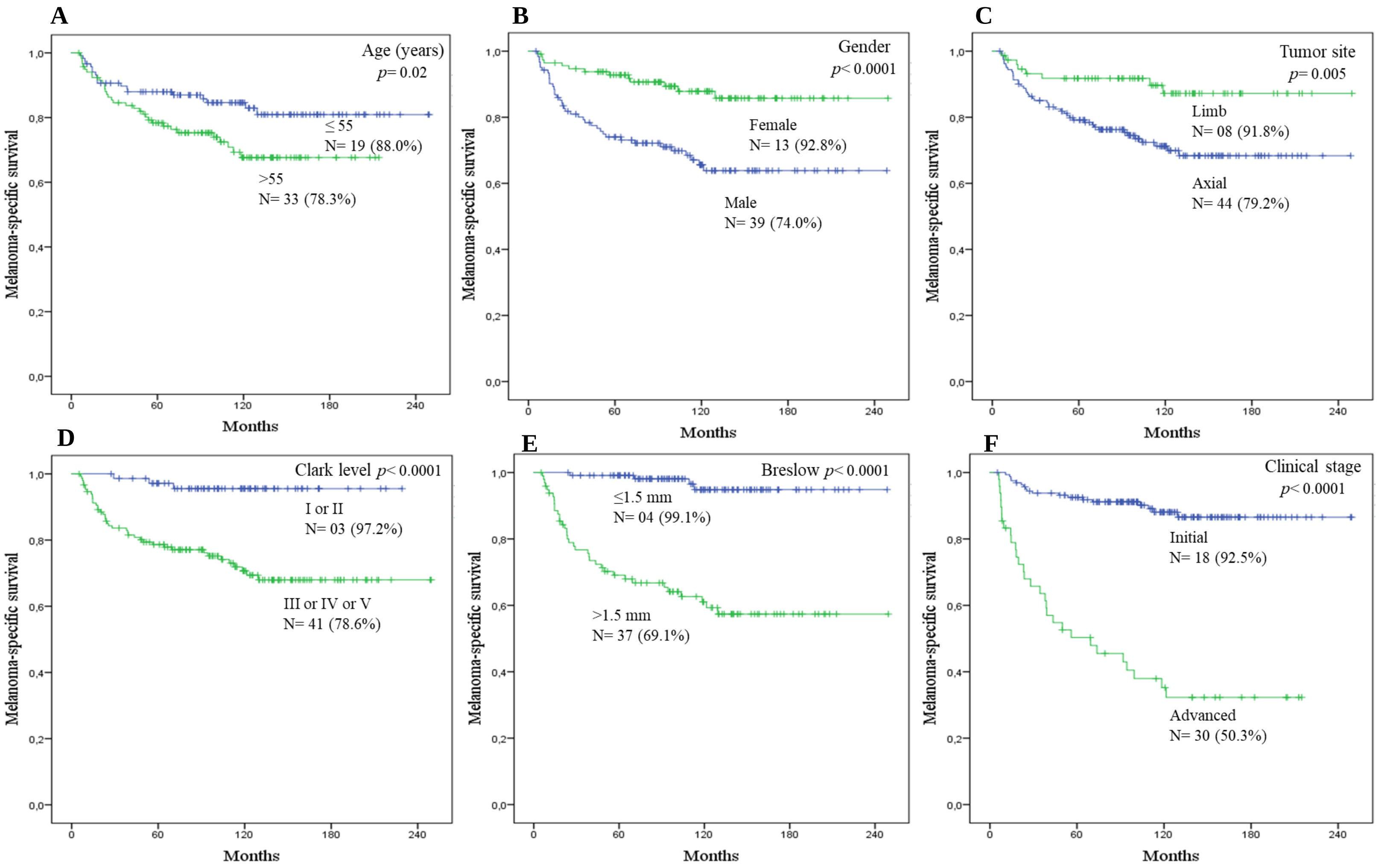

Supplementary Figure S3

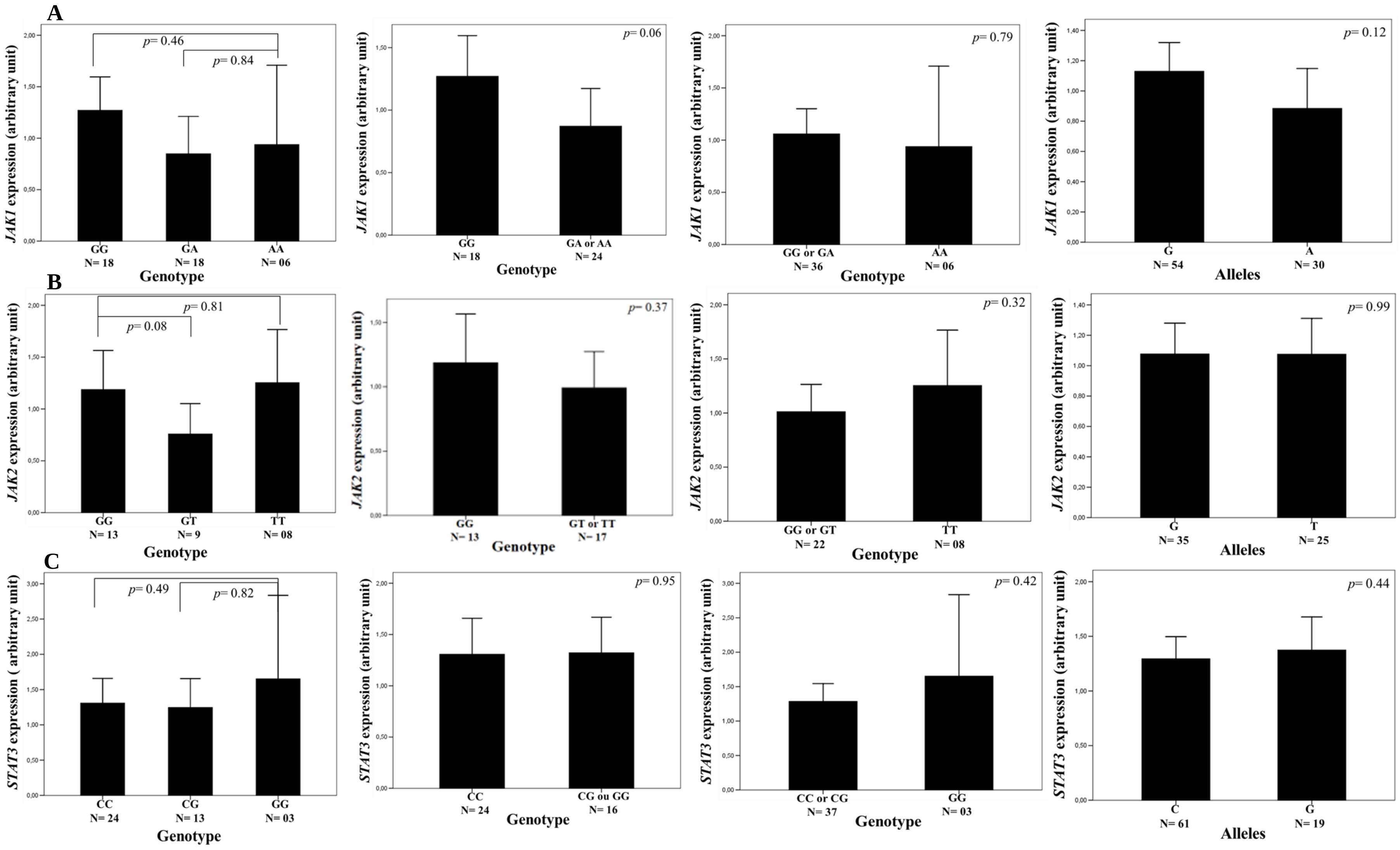

Supplementary Figure S4

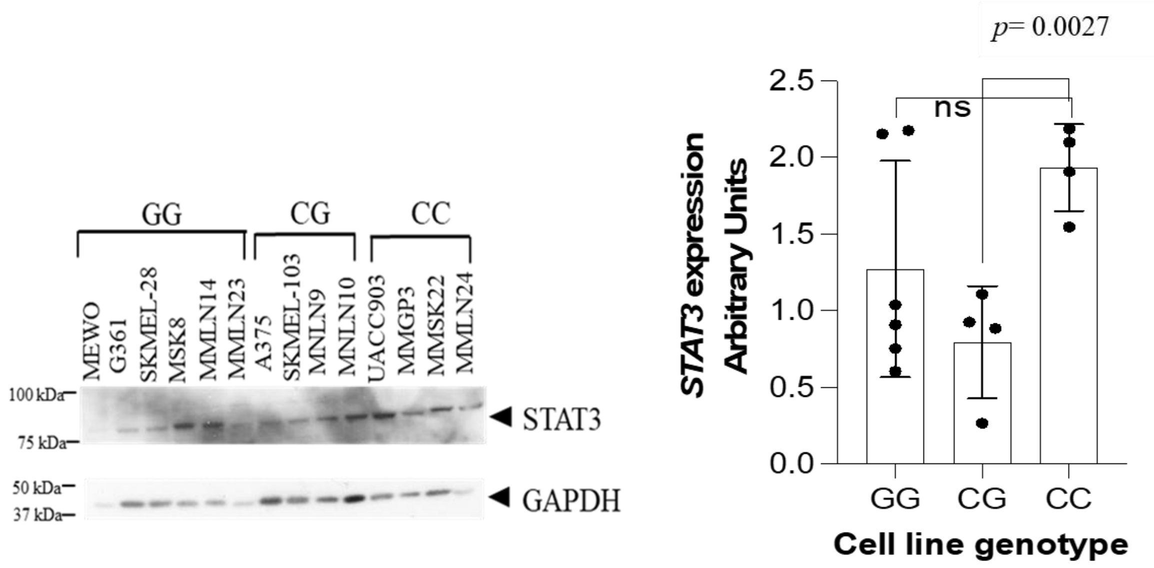

Supplementary Figure S5

Supplementary Figure S1. Steps for selecting single nucleotide variants (SNVs) for the study. First, SNVs that have previously been associated with cancer risk and/or survival of cancer patients were selected from public database. After, SNVs that had more studies in different types of cancer were filtered according to the minor allele frequency ≥ 10% in HapMap global population and sample size calculation. Finally, *in silico* analysis with Variant Effect Predictor (VEP), Human Splicing Finder, Genomatrix, SNPinfo, MicroSNiPer and MirSNPscore programs was performed with the purpose of to find SNVs with high biological plausibility of being involved in tumor origin or progression. At the end, *JAK1* c.1648+1272G>A (rs310211), *JAK1* c.991-27C>T (rs2256298), *JAK2* c.-1132G>T (rs1887429), *JAK2* c.-139G>A (rs2274472), *STAT3* c.*1671T>C (rs1053004), and *STAT3* c.-1937C>G (rs4796793) were selected for study

Supplementary Figure S2. Kaplan-Meier plots indicating lower rates of progression-free survival in cutaneous melanoma male patients (**A**), patients with axial tumor (**B**), vertically growing tumor (**C**), tumor with Clark levels III to V (**D**), Breslow index higher than 1.5 mm (**E**), and at III or IV stage (**F**). For statistical tests, values with $p < 0.05$ were considered significant.

Supplementary Figure S3. Kaplan-Meier plots indicating lower rates of melanoma specific survival in cutaneous melanoma older patients (**A**), male patients (**B**), patients with axial tumor (**C**), tumor with Clark levels III to V (**D**), Breslow index higher than 1.5 mm (**E**), and at III or IV stage (**F**) For statistical tests, values with $p < 0.05$ were considered significant.

Supplementary Figure S4. Expression of the *JAK1*, *JAK2* and *STAT3* genes in leukocytes of peripheral blood samples cutaneous melanoma patients measured by real-time polymerase chain reaction. The relative expression level was normalized by β-actin (BAC) reference gene, with the 2^{-DDCt} cycle threshold method. Values of 15% of the samples were repeated in separate experiments with 100% agreement and results were expressed in arbitrary units (AUs). For statistical tests, values with $p < 0.05$ were considered significant. Similar mean values of *JAK1* expression were found in patients with distinct genotypes of the *JAK1* c.1648+1272G>A (**A**), *JAK2* c.-1132G>T (**B**), and *STAT3* c.-1937C>G single nucleotide variants (**C**)

Supplementary Figure S5. STAT3 protein level in different melanoma cell lines grouped by CC, CG and GG genotypes of *STAT3* c.-1937C>G single nucleotide variant by western blot using endogenous glyceraldehyde-3-phosphate dehydrogenase (GAPDH) normalizers. MSK8, MMLN14, MMLN23, MMLN9, MMLN10, MMGP3, MMSK22 and MMLN24 are patient-derived cells and the others are commercial cells. Three independent experiments were performed. ECL Western Blot Detection Reagents kit (GE Healthcare, Bethesda, MD, USA) was used and the intensities of protein bands were quantitated using ImageJ software (National Institutes of Health, USA). For statistical tests, values with $p < 0.05$ were considered significant. Cells with CC genotype presented higher STAT3 protein level than those with GG genotype (1.93 *versus* 1.27 arbitrary units (AUs), $p = 0.0027$)
