# Supplementary material for: Association of JAK/STAT genetic variants with cutaneous melanoma
Source: Front Oncol. 2022 Aug 2;12:943483. doi: 10.3389/fonc.2022.943483 (PMC9379289; doi:10.3389/fonc.2022.943483)
Supplement: Supplementary file 2 [file Table_1.doc]

**Supplementary Table 1**. Single nucleotide variants in *JAK1, JAK2* and *STAT3* genes selection for study

| **SNV** | **Gene** | **Localization** | **MAF (%)** | **Cancer** | **Authors** |
| --- | --- | --- | --- | --- | --- |
| rs10889513 | *JAK1* | Promoter | 16 | Gastric | Zhou *et al*., 2016 |
| rs2230587 | *JAK1* | Missense variant | 13 | Gastric | Zhou *et al*., 2016 |
| rs2254002 | *JAK1* | Intron | 47 | Breast | Slattery *et al*., 2014 |
| rs2256298* | *JAK1* | Intron | 27 | Breast, colorectal | Slattery *et al.,* 2013, Slattery *et al.,* 2014 |
| rs2780890 | *JAK1* | Intron | 48 | Breast | Slattery *et al.,* 2014 |
| rs310198 | *JAK1* | Intron | 16 | Colorectal | Slattery *et al*., 2013 |
| rs310199 | *JAK1* | Intron | 32 | Colorectal | Slattery *et al*., 2013 |
| rs310211* | *JAK1* | Intron | 18 | Breast, colorectal | Slattery *et al*., 2013, Slattery *et al.,* 2014 |
| rs310245 | *JAK1* | Intron | 40 | Breast | Slattery *et al.,* 2014 |
| rs3790541 | *JAK1* | Intron | 12 | Colorectal | Slattery *et al*., 2013 |
| rs4916005 | *JAK1* | Intron | 15 | Breast | Slattery *et al.,* 2014 |
| rs10815160* | *JAK2* | Intron | 31 | Breast, colorectal | Slattery *et al*., 2013, Slattery *et al.*, 2014, Zhang *et al.,* 2015 |
| rs10974916 | *JAK2* | Intron | 32 | Breast | Slattery *et al.,* 2014 |
| rs10974947* | *JAK2* | Intron | 25 | Breast, colorectal | Slattery *et al.,* 2013, Slattery *et al.,* 2014, Zhang *et al.,* 2015 |
| rs11793659 | *JAK2* | Intron | 28 | Breast | Vaclavicek *et al.*, 2007 |
| rs1410779 | *JAK2* | Intron | 19 | Breast | Vaclavicek *et al.*, 2007 |
| rs1536800 | *JAK2* | Intron | 23 | Breast | Slattery *et al.,* 2014 |
| rs1887428 | *JAK2* | Promoter | 47 | Breast | Vaclavicek *et al.*, 2007 |
| rs1887429* | *JAK2* | Promoter | 28 | Breast, colorectal, gastric | Vaclavicek et al., 2007, Slattery *et al*., 2013, Slattery *et al.,* 2014, Zhang *et al.,* 2015, Zhou *et al*., 2016 |
| rs2230722 | *JAK2* | Promoter | 33 | Breast | Vaclavicek *et al.*, 2007 |
| rs2230724 | *JAK2* | Intron | 49 | Breast | Vaclavicek *et al.*, 2007 |
| rs2239723 | *JAK2* | Intron | 36 | Breast | Vaclavicek *et al.*, 2007 |
| rs2274471* | *JAK2* | Promoter | 24 | Breast, colorectal | Vaclavicek *et al.*, 2007, Slattery *et al.,* 2013, Slattery *et al.,* 2014 |
| rs2274472* | *JAK2* | Promoter | 30 | Breast, colorectal | Vaclavicek *et al.*, 2007, Slattery *et al.,* 2013, Slattery *et al.,* 2014 |
| rs3780379* | *JAK2* | Intron | 20 | Breast, colorectal | Slattery *et al*., 2013, Slattery *et al.,* 2014, Zhang *et al*., 2015 |
| rs3780381 | *JAK2* | Intron | 28 | Colorectal | Slattery *et al*., 2013 |
| rs3808850* | *JAK2* | Promoter | 19 | Breast, gastric | Vaclavicek *et al.,* 2007, Zhou *et al*., 2016 |
| rs6476933 | *JAK2* | Promoter | 29 | Gastric | Zhou *et al*., 2016 |
| rs7034539 | *JAK2* | Intron | 24 | Breast | Vaclavicek *et al.*, 2007 |
| rs7034753 | *JAK2* | Intron | 42 | Breast | Vaclavicek *et al.*, 2007 |
| rs7043371* | *JAK2* | Intron | 42 | Breast, colorectal | Slattery *et al.,* 2013, Slattery *et al.*, 2014 |
| rs780381 | *JAK2* | Intron | 11 | Breast | Slattery *et al.,* 2014 |
| rs1026916* | *STAT3* | Intron | 36 | Breast, colorectal | Slattery *et al.,* 2013, Slattery *et al.,* 2014 |
| rs1053004* | *STAT3* | 3'UTR | 38 | Gastric, hepatocellular carcinoma, non-small cell lung cancer, pancreatic | Jiang *et al.,* 2011, Xie *et al.,* 2013, Chanthra *et al*., 2015, Zhou *et al*., 2016, Zhu *et al.,* 2016, |
| rs1053005* | *STAT3* | 3'UTR | 21 | Breast, colorectal, gastric, Hodgkin lymphoma, pancreatic | Butterbach *et al*., 2011, Slattery *et al*., 2013, Slattery *et al*., 2014, Zhu *et al.,* 2016, Zhou *et al*., 2016 |
| rs1053023 | *STAT3* | 3'UTR | 13 | Hodgkin lymphoma | Butterbach *et al.*, 2011 |
| rs12949918* | *STAT3* | Intron | 40 | Breast, cancer risk: meta-analysis, colorectal, Hodgkin lymphoma, non-small cell lung cancer | Butterbach *et al.,* 2011, Jiang *et al*., 2011, Slattery *et al.,* 2013, Slattery *et al.,* 2014, Yan *et al.,* 2015, Zhang *et al.,* 2015 |
| rs2293152* | *STAT3* | Intron | 41 | Breast, cancer risk: meta-analysis, colorectal, hepatocellular carcinoma, non-small cell lung cancer | Vaclavicek *et al.*, 2007, Jiang *et al.,* 2011, Slattery *et al.,* 2013, Xie *et al.,* 2013, Yan *et al*., 2015, Zhang *et al.,* 2015 |
| rs2306581 | *STAT3* | Intron | 43 | Breast | Vaclavicek *et al.*, 2007 |
| rs34997637 | *STAT3* | Intron | 24 | Hodgkin lymphoma | Butterbach *et al.*, 2011 |
| rs3869550 | *STAT3* | Intron | 43 | Non-small cell lung cancer | Jiang *et al.*, 2011 |
| rs4103200 | *STAT3* | Intron | 27 | Hodgkin lymphoma | Butterbach *et al.*, 2011 |
| rs4796793* | *STAT3* | Promoter | 35 | Breast, cancer risk: meta-analysis, cervical, colorectal, gastric, hepatocellular carcinoma, melanoma, metastatic renal cell carcinoma, non-small cell lung | Ito *et al.,* 2007, Jiang *et al.,* 2011, Wang *et al.,* 2011, Xie *et al.,* 2013, Schrama *et al.*, 2014, Yan *et al.,* 2015, Zhao *et al.,* 2015, Yamamoto *et al*., 2016, Zhou *et al.,* 2016 |
| rs6503695* | *STAT3* | Intron | 34 | Breast, cancer risk: meta-analysis, colorectal, Hodgkin lymphoma, non-small cell lung cancer | Butterbach *et al.*, 2011, Slattery *et al*., 2013, Slattery *et al.,* 2014, Yan *et al.,* 2015, Zhao *et al*., 2015 |
| rs7211777* | *STAT3* | Intron | 40 | Breast | Vaclavicek *et al.*, 2007, Jiang *et al.,* 2011 |
| rs744166* | *STAT3* | Intron | 42 | Cancer risk: meta-analysis, colorectal, non-small cell lung cancer | Jiang *et al.,* 2011, Yan *et al.,* 2015, Zhang *et al.*, 2015, Matsusaka *et al*., 2016 |
| rs8069645 | *STAT3* | Intron | 28 | Breast | Slattery *et al.,* 2014 |
| rs9891119 | *STAT3* | Intron | 36 | Hodgkin lymphoma | Butterbach *et al.*, 2011 |
| rs9912773* | *STAT3* | Intron | 13 | Hodgkin lymphoma, non-small cell lung cancer | Butterbach *et al.*, 2011, Jiang *et al.,* 2011 |

SNV: single nucleotide variant; MAF: minor allele frequency; 3'UTR: 3′ untranslated region. * SNVs that had more studies in different types of cancer and MAF > 10%

Data was obtained from dbSNP database (http://www.ncbi.nlm.nih.gov/projects/SNP)

**References**

Butterbach K, Beckmann L, Sanjose S, Benavente Y, Becker N, Foretova L, Maynadie M, Cocco P, Staines A, Boffetta P, Brennan P, Nieters A (2011). Association of JAKSTAT pathway related genes with lymphoma risk: results of a European case–control study (EpiLymph). British Journal of Haematology, 153:318–333.

Chanthra N, Payungporn S, Chuaypen N, Pinjaroen N, Poovorawan Y, Tangkijvanich P (2015). Association of Single Nucleotide Polymorphism rs1053004 in Signal Transducer and Activator of Transcription 3 (STAT3) with Susceptibility to Hepatocellular Carcinoma in Thai Patients with Chronic Hepatitis B. Asian Pacific Journal of Cancer Prevention, 16:5069-5073.

Ito N, Eto M, Nakamura E, Takahashi A, Tsukamoto T, Toma H, Nakazava H, Hirao Y, Uemura H, Kagawa S, Kanayama H, Nose Y, Kinukawa N, Nakamura T, Jinnai N, Seki T,Tamatsu M, Masui Y, Naito S, Ogawa O (2007). STAT3 polymorphism predicts interferon-alfa response in patients with metastatic renal cell carcinoma. Journal of Clinical Oncology, 25:2785-2791.

Jiang B, Zhu Z.Z, Liu F, Yang L.J, Zhang W.Y, Yuan H.H, Wang J.G,Hu X.H., Huang G (2011). STAT3 gene polymorphisms and susceptibility to non-small cell lung cancer. Genetics and Molecular Research, 10:1856-1865.

Matsusaka S, Hanna DL, Cao S, Zhang W, Yang D, Ning Y, Sunakawa Y, Okazaki S, Berger MD, Miyamato Y, Parekh A, Stintzing S, Loupakis F, Lenz H (2016). Prognostic impact of IL6 genetic variants in patients with metastatic colorectal câncer treated with bevacizumab-based chemotherapy. Clinical Cancer Research, 2:1-9.

Schrama D, Ugurel S, Sucker A, Ritter C, Zapatka M, Schadendorf S, Becker JC (2014). STAT3 single nucleotide polymorphism rs4796793 SNP does not correlate with

response to adjuvant IFNa therapy in stage III melanoma patients. Frontiers in Medicine, 1:1-6.

Slattery M.L, Lundgreen A, Kadlubar SA, Bondurant KL, Wolff RK (2013). JAK/STAT/SOCS-signaling pathway and colon and rectal cancer. Molecular Carcinogenesis, 52:155-166.

Slattery ML, Lundgreen A, Hines LM, Torres-Mejia G, Wolff RK, Stern MC, John EM (2014). Genetic variation in the JAK/STAT/SOCS signaling pathway influences breast cancer-specific mortality through interaction with cigarette smoking and use of aspirin/NSAIDs: The Breast Cancer Health Disparities Study. Breast Cancer Research Treatment, 147:145-158.

Vaclavicek A, Bermejo JL, Schmutzler RK, Sutter C, Wappenschmidt B, Meindl A, Kiechle M, Arnold N, Weber BHF, Niederacher D, Burwinkel B, Bartram CR, Hemminki K, Forsti A (2007). Polymorphisms in the Janus kinase 2 (JAK)/ signal transducer and activator of transcription (STAT) genes: putative association of the STAT gene region with familial breast cancer. Endocrine-Related Cancer, 14:267– 277.

Wang K, Zhou B, Zhang J, Xin Y, Lai T, Wang Y, Hou Q, Song Y, Chen Y, Quan Y, Xi M, Zhang L (2011). Association of Signal Transducer and Activator of Transcription 3 Gene Polymorphisms with Cervical Cancer in Chinese Women. DNA and Cell Biology, 30:931-936.

Xie J, Zhang Y, Zhang Q, Han Y, Yin J, Pu R, Shen Q, Lu W, Du Y (2013). Interaction of signal transducer and activator of transcription 3 polymorphisms with hepatitis b virus mutations in hepatocellular carcinoma. Hepatology, 5:2369-2377.

Yamamoto K, Ioroi T, Kanaya K, Shinomiya K, Komoto S, Hirata S, Harada K, Watanabe A, Sumo M, Nishioka T, Kume M, Makimoto H, Nakagawa T, Hirano T, Miyake H, Fujisawa M, Hirai M (2016). STAT3 polymorphism rs4796793 may be a predictive factor of tumor response to multiple tyrosine kinase inhibitors in metastatic renal cell carcinoma in Japanese population. Medical Oncology, 33:24-31.

Yan R, Lin F, Hu C, Tong S (2015). Association between STAT3 polymorphisms and cancer risk: a meta-analysis. Molecular Genetics and Genomics, 290:2261-2270.

Zhao L, Zhang Q, Luan X, Huang X, Zhao S, Zhao H (2015). STAT3 and STAT5b polymorphism contributes to breast cancer risk and clinical outcomes. International Journal of Clinical Experiment, 8:2033-2038.

Zhou F, Cheng L, Qiu L, Wang M, Li J Sun M, Yang Y, Wang J, Jin L, Wang Y, Wei Q (2016). Associations of potentially functional variants in IL-6, JAKs and STAT3 with gastric cancer risk in an eastern Chinese population. Oncotarget, 7:28112-28123.

Zhu Z, Liu W, Gotlieb V (2016). The rapidly evolving therapies for advanced melanoma- Towards immunotherapy, molecular targeted therapy, and beyond. Critical Reviews in Oncology/Hematology, 99:91-99.

**Supplementary Table 2**. *In silico* analysis for selection of single nucleotide variants (SNV) in *JAK1, JAK2* and *STAT3* genes

| **SNV** | **Gene** | **Localization** | **MAF (%)** | **Biological consequence** |
| --- | --- | --- | --- | --- |
| rs2256298* | *JAK1* | Intron | 27 | Splicing region |
| rs310211* | *JAK1* | Intron | 18 | Splicing region |
| rs10815160 | *JAK2* | Intron | 31 | Splicing region |
| rs10974947 | *JAK2* | Intron | 25 | Splicing region |
| rs1887429* | *JAK2* | Promoter | 28 | The variant allele has gain linking site of XBBF factor transcript |
| rs2274471 | *JAK2* | Promoter | 24 | Transcription factor binding |
| rs2274472* | *JAK2* | Promoter | 30 | The variant allele has gain binding site of XCPE, ZTRE and E2FF factor transcription |
| rs3780379 | *JAK2* | Intron | 20 | No consequence |
| rs3808850 | *JAK2* | Promoter | 19 | Transcription factor binding |
| rs7043371 | *JAK2* | Intron | 42 | No consequence |
| rs1026916 | *STAT3* | Intron | 36 | Splicing region |
| rs1053004* | *STAT3* | 3'UTR | 38 | The variant allele promotes increased and reduced binding to the miR-610 and miR-31-5p microRNA, respectively |
| rs1053005 | *STAT3* | 3'UTR | 21 | No consequence |
| rs12949918 | *STAT3* | Intron | 40 | Splicing region |
| rs2293152 | *STAT3* | Intron | 41 | Splicing region |
| rs4796793* | *STAT3* | Promoter | 35 | The variant allele has loss the linking site of the FKHD and LEFF factors transcription |
| rs6503695 | *STAT3* | Intron | 34 | Splicing region |
| rs7211777 | *STAT3* | Intron | 40 | Splicing region |
| rs744166 | *STAT3* | Intron | 42 | Splicing region |
| rs9912773 | *STAT3* | Intron | 13 | Splicing region |

SNV: single nucleotide variant; MAF: minor allele frequency; 3'UTR: 3′ untranslated region; * SNVs selected for the study

**Supplementary Table 3. Specific primers used for gene expression and promoter region activity measurements**

| **Gene** | **Direction** | **Primer** |
| --- | --- | --- |
| ***JAK1*, *JAK2*, and *STAT3* expression in peripheral blood samples** | | |
| *JAK1* | Forward | 5’-TCCCATGGCTTTGTTCCTG-3’ |
|  | Reverse | 5’-CCCACCATTTCCTCATAAGTTG-3’ |
| *JAK2* | Forward | 5’-TTCATCCAGCCATGTTATCCC-3’ |
|  | Reverse | 5’-TGGAAGATTACCAAGACCAGATG-3’ |
| *STAT3* | Forward | 5’-ACTAAAGTCAGGTTGCTGGTC-3’ |
|  | Reverse | 5’-TTTGTGTTTGTGCCCAGAATG-3’ |
| β-actin | Forward | 5’-AGGCCAACCGCGAGA-AG-3’ |
|  | Reverse | 5’-ACAGC-CTGGATAG-CAACGTACA-3’ |
| ***STAT3* promoter region activity in modified SKMEL-28** **cell line** | | |
| *STAT3* | Forward | 5’-TTGGTACCCCTGACTCTGAACCTG-3’ |
|  | Reverse | 5’-CGCGCCATGGTGCTAAAATCAGGGGTCC-3’ |
| ***STAT3* expression in modified SKMEL-28** **cell line** | | |
| *STAT3* | Forward | 5’-CGCGCCATGGCCCAAT-GGAATCAG-3’ |
|  | Reverse | 5’-GCGCTCTAGAATCACATGGGGGAGGTAG-3’ |

**Supplementary Table 4. Description of the genetic profile of the 14 melanoma cell lines**

| **Cell line** | **Origin** | **Mutation** | ***STAT3* c.-1937C>G genotype** |
| --- | --- | --- | --- |
| G361 | Commercial cells | *NRAS* and *BRAF* mutated | GG |
| A375 | Commercial cells | *NRAS* and *BRAF* mutated | CG |
| SKMEL-28 | Commercial cells | *BRAF* mutated | GG |
| SKMEL-103 | Commercial cells | *NRAS* e *BRAF* mutated | CG |
| MSK8 | Patient-derived cells | NA | GG |
| MMLN9 | Patient-derived cells | *NRAS* mutated | CG |
| MMLN10 | Patient-derived cells | *NRAS* mutated | CG |
| MMLN14 | Patient-derived cells | NA | GG |
| MMLN23 | Patient-derived cells | NA | GG |
| MMGP3 | Patient-derived cells | NA | CC |
| MMSK22 | Patient-derived cells | NA | CC |
| MMLN24 | Patient-derived cells | NA | CC |
| UACC903 | Commercial cells | *BRAF* and *NRAS* mutated | CC |
| MEWO | Commercial cells | *BRAF* and *NRAS* not mutated | GG |

NA: not analyzed

**Supplementary Table 5**. *JAK1*, *JAK2* and *STAT3* combined genotypes in 248 cutaneous melanoma patients and 274 controls

| **Genotype** | **Patients**  **N (%)** | **Controls  N (%)** | ***p-*value** | **OR* (95% CI)** |
| --- | --- | --- | --- | --- |
| ***JAK1* c.1648+1272G>A + *JAK1* c.991-27C>T** |  |  |  |  |
| GG + CC | 99 (44.0) | 100 (39.1) | 0.26 | Reference |
| GA or AA + CT or TT | 126 (56.0) | 156 (60.9) |  | 1.31 (0.81-2.11) |
| GG or GA + CC or CT | 205 (88.0) | 230 (85.2) | 0.95 | Reference |
| AA + TT | 28 (12.0) | 40 (14.8) |  | 1.01 (0.53-1.94) |
| ***JAK1* c.1648+1272G>A + *JAK2* c.-1132G>T** |  |  |  |  |
| GG + GG | 49 (35.5) | 47 (33.3) | 0.71 | Reference |
| GA or AA + GT or TT | 89 (64.5) | 94 (66.7) |  | 1.12 (0.60-2.08) |
| GG or GA + GG or GT | 180 (95.7) | 199 (97.1) | 0.10 | Reference |
| AA + TT | 08 (4.3) | 06 (2.9) |  | 2.95 (0.79-10.94) |
| ***JAK1* c.1648+1272G>A + *JAK2* c.-139G>A** |  |  |  |  |
| GG + GG | 96 (99.0) | 100 (99.0) | 0.84 | Reference |
| GA or AA + GA or AA | 01 (1.0) | 01 (1.0) |  | 1.70 (0.007-420.98) |
| GG or GA + GG or GA | 205 (100.0) | 230 (100.0) | NC | Reference |
| AA + AA | 0 (0.0) | 0 (0.0) |  | NC |
| ***JAK1* c.1648+1272G>A + *STAT3*c.*1671T>C** |  |  |  |  |
| GG + TT | 38 (27.9) | 29 (19.5) | **0.01** | 2.54 (1.23-5.25) |
| GA or AA + TC or CC | 98 (72.1) | 120 (80.5) |  | Reference |
| GG or GA + TT or TC | 182 (88.3) | 208 (86.7) | 0.72 | 1.13 (0.55-2.32) |
| AA + CC | 24 (11.7) | 32 (13.3) |  | Reference |
| ***JAK1* c.1648+1272G>A + *STAT3* c.-1937C>G** |  |  |  |  |
| GG + CC | 55 (43.0) | 44 (31.0) | **0.02** | 2.15 (1.12-4.13) |
| GA or AA + CG or GG | 73 (57.0) | 98 (69.0) |  | Reference |
| GG or GA + CC or CG | 188 (97.4) | 204 (97.6) | 0.12 | 3.45 (0.71-16.63) |
| AA + GG | 05 (2.6) | 05 (2.4) |  | Reference |
| ***JAK1* c.991-27C>T + *JAK2* c.-1132G>T** |  |  |  |  |
| CC + GG | 55 (43.3) | 59 (40.1) | 0.88 | Reference |
| CT or TT + GT or TT | 72 (56.7) | 88 (59.9) |  | 1.04 (0.56-1.93) |
| CC or CT + GG or GT | 193 (97.0) | 202 (97.6) | 0.09 | Reference |
| TT + TT | 06 (3.0) | 05 (2.4) |  | 3.44 (0.80-14.75) |
| ***JAK1* c.991-27C>T + *JAK2* c.-139G>A** |  |  |  |  |
| CC + GG | 119 (99.2) | 118 (99.2) | 0.83 | Reference |
| CT or TT + GA or AA | 01 (0.8) | 01 (0.8) |  | 1.70 (0.01-277.47) |
| CC or CT + GG or GA | 248 (100.0) | 274 (100.0) | NC | Reference |
| TT + AA | 0 (0.0) | 0 (0.0) |  | NC |
| ***JAK1* c.991-27C>T + *STAT3* c.*1671T>C** |  |  |  |  |
| CC + TT | 46 (35.7) | 34 (24.1) | **0.01** | 2.32 (1.16-4.64) |
| CT or TT + TC or CC | 83 (64.3) | 107 (75.9) |  | Reference |
| CC or CT + TT or TC | 183 (96.8) | 185 (92.0) | 0.46 | 1.49 (0.51-4.35) |
| TT + CC | 06 (3.2) | 16 (8.0) |  | Reference |
| ***JAK1* c.991-27C>T + *STAT3* c.-1937C>G** |  |  |  |  |
| CC + CC | 67 (51.9) | 52 (37.1) | **0.02** | 2.10 (1.10-4.00) |
| CT or TT + CG or GG | 62 (48.1) | 88 (62.9) |  | Reference |
| CC or CT + CC or CG | 200 (99.0) | 208 (97.7) | 0.48 | 1.97 (0.28-13.68) |
| TT + GG | 02 (1.0) | 05 (2.3) |  | Reference |
| ***JAK2* c.-1132G>T + *JAK2* c.-139G>A** |  |  |  |  |
| GG + GG | 108 (97.3) | 126 (100.0) | 0.99 | Reference |
| GT or TT + GA or AA | 03 (2.7) | 0 (0.0) |  | NC |
| GG or GT + GG or GA | 215 (100.0) | 237 (100.0) | NC | Reference |
| TT + AA | 0 (0.0) | 0 (0.0) |  | NC |
| ***JAK2 c.-*1132G>T + *STAT3* c.*1671T>C** |  |  |  |  |
| GG + TT | 39 (30.5) | 39 (27.5) | 0.51 | 1.24 (0.64-2.43) |
| GT or TT + TC or CC | 89 (69.5) | 103 (72.5) |  | Reference |
| GG or GT + TT or TC | 181 (95.3) | 178 (96.7) | 0.23 | 2.48 (0.55-11.16) |
| TT + CC | 09 (4.7) | 06 (3.3) |  | Reference |
| ***JAK2* c.-1132G>T + *STAT3* c.-1937C>G** |  |  |  |  |
| GG + CC | 56 (46.7) | 59 (40.7) | 0.49 | Reference |
| GT or TT + CG or GG | 64 (53.3) | 86 (59.3) |  | 1.24 (0.66-2.32) |
| GG or GT + CC or CG | 198 (97.5) | 207 (99.5) | 0.06 | Reference |
| TT + GG | 05 (2.5) | 01 (0.5) |  | 1.57 (1.38-8.67) |
| ***JAK2 c.-*139G>A + *STAT3* c.*1671T>C** |  |  |  |  |
| GG + TT | 87 (97.8) | 82 (100.0) | 0.99 | NC |
| GA or AA + CT or CC | 02 (2.2) | 0 (0.0) |  | Reference |
| GG or GA + TT or CT | 205 (100.0) | 209 (100.0) | NC | NC |
| AA + CC | 0 (0.0) | 0 (0.0) |  | Reference |
| ***JAK2 c.-*139G>A + *STAT3* c.-1937C>G** |  |  |  |  |
| GG + CC | 128 (99.2) | 119 (100.0) | 1.00 | NC |
| GA or AA + CG or GG | 01 (0.8) | 0 (0.0) |  | Reference |
| GG or GA + CC or CG | 226 (100.0) | 243 (100.0) | NC | NC |
| AA + GG | 0 (0.0) | 0 (0.0) |  | Reference |
| ***STAT3* c.*1671T>C + *STAT3* c.-1937C>G** |  |  |  |  |
| TT + CC | 82 (42.7) | 74 (33.8) | **0.01** | 1.90 (1.13-3.19) |
| TC or CC + CG or GG | 110 (57.3) | 145 (66.2) |  | Reference |
| TT or TC + CC or CG | 201 (91.8) | 202 (89.4) | 0.70 | 1.17 (0.51-2.65) |
| CC + GG | 18 (8.2) | 24 (10.6) |  | Reference |
| ***JAK1* c.1648+1272G>A + *JAK1* c.991-27C>T + *JAK2* c.-1132G>T** |  |  |  |  |
| GG + CC + GG | 49 (40.5) | 47 (34.8) | 0.47 | Reference |
| GA or AA + CT or TT + GT or TT | 72 (59.5) | 88 (65.2) |  | 1.26 (0.66-2.39) |
| GG or GA + CC or CT + GG or GT | 180 (96.8) | 199 (97.5) | 0.08 | Reference |
| AA + TT + TT | 06 (3.2) | 05 (2.5) |  | 3.56 (0.83-15.27) |
| ***JAK1* c.1648+1272G>A + *JAK1* c.991-27C>T + *JAK2* c.-139G>A** |  |  |  |  |
| GG + CC + GG | 96 (99.0) | 100 (99.0) | 0.84 | Reference |
| GA or AA + CT or TT + GA or AA | 01 (1.0) | 01 (1.0) |  | 1.70 (0.007-420.98) |
| GG or GA + CC or CT + GG or GA | 205 (100.0) | 230 (100.0) | NC | Reference |
| AA + TT + AA | 0 (0.0) | 0 (0.0) |  | NC |
| ***JAK1* c.1648+1272G>A + *JAK1* c.991-27C>T + *STAT3* c.*1671T>C** |  |  |  |  |
| GG + CC + TT | 38 (31.4) | 29 (21.3) | **0.01** | 2.66 (1.25-5.62) |
| GA or AA + CT or TT +TC or CC | 83 (68.6) | 107 (78.7) |  | Reference |
| GG or GA + CC or CT + TT or TC | 172 (96.6) | 183 (92.0) | 0.51 | 1.42 (0.49-4.17) |
| AA + TT + CC | 06 (3.4) | 16 (8.0) |  | Reference |
| ***JAK1* c.1648+1272G>A + *JAK1* c.991-27C>T + *STAT3* c.-1937C>G** |  |  |  |  |
| GG + CC + CC | 55 (47.0) | 44 (33.3) | **0.01** | 2.35 (1.18-4.67) |
| GA or AA + CT or TT + CG or GG | 62 (53.0) | 88 (66.7) |  | Reference |
| GG or GA + CC or CT + CC or CG | 188 (98.9) | 204 (97.6) | 0.47 | 2.02 (0.29-14.12) |
| AA + TT + GG | 02 (1.1) | 05 (2.4) |  |  |
| ***JAK1* c.991-27C>T + *JAK2* c.-1132G>T + *STAT3* c.*1671T>C** |  |  |  |  |
| CC + GG + TT | 21 (30.9) | 15 (19.2) | 0.10 | 2.23 (0.85-5.83) |
| CT or TT + GT or TT + TC or CC | 47 (69.1) | 63 (80.8) |  | Reference |
| CC or CT + GG or GT + TT or TC | 163 (98.8) | 158 (99.4) | 0.36 | 3.33 (0.25-44.04) |
| TT + TT + CC | 02 (1.2) | 01 (0.6) |  | Reference |
| ***JAK1* c.991-27C>T + *JAK2* c.-1132G>T + *STAT3* c.-1937C>G** |  |  |  |  |
| CC + GG + CC | 31 (48.4) | 28 (35.4) | 0.09 | 2.16 (0.88-5.28) |
| CT or TT + GT or TT + CG or GG | 33 (51.6) | 51 (64.6) |  | Reference |
| CC or CT + GG or GT + CC or CG | 177 (99.4) | 177 (100.0) | 1.00 | NC |
| TT + TT + GG | 01 (0.6) | 0 (0.0) |  | Reference |
| ***JAK2* c.-1132G>T + *JAK2* c.-139G>A + *STAT3* c.*1671T>C + *STAT3* c.-1937C>G** |  |  |  |  |
| GG + GG + TT + CC | 36 (37.5) | 31 (27.0) | 0.21 | 1.58 (0.76-3.25) |
| GT or TT + GA or AA + TC or CC + CG or GG | 60 (62.5) | 84 (73.0) |  | Reference |
| ***JAK1* c.1648+1272G>A + *JAK1* c.991-27C>T + *JAK2* c.-1132G>T + *JAK2* c.-139G>A** |  |  |  |  |
| GG + CC + GG + GG | 48 (31.4) | 47 (26.3) | 0.26 | Reference |
| GA or AA + CT or TT + GT or TT + GA or AA | 105 (68.6) | 132 (73.7) |  | 1.42 (0.77-2.62) |
| ***JAK1* c.1648+1272G>A + *JAK1* c.991-27C>T + *JAK2* c.-1132G>T + *STAT3* c.*1671T>C** |  |  |  |  |
| GG + CC + GG + TT | 19 (28.8) | 11 (14.9) | **0.02** | 3.56 (1.22-10.37) |
| GA or AA + CT or TT + GT or TT + TC or CC | 47 (71.2) | 63 (85.15) |  | Reference |
| ***JAK1* c.1648+1272G>A + *JAK1* c.991-27C>T + *JAK2* c.-1132G>T + *STAT3* c.-1937C>G** |  |  |  |  |
| GG + CC + GG + CC | 28 (45.9) | 21 (29.2) | **0.01** | 3.21 (1.22-8.47) |
| GA or AA + CT or TT + GT or TT + CG or GG | 33 (54.1) | 51 (70.8) |  | Reference |
| ***JAK1* c.1648+1272G>A + *JAK1* c.991-27C>T + *STAT3* c.*1671T>C + *STAT3* c.-1937C>G** |  |  |  |  |
| GG + CC + TT + CC | 34 (36.6) | 24 (22.2) | **0.002** | 3.95 (1.66-8.95) |
| GA or AA +CT or TT + TC or CC + CG or GG | 59 (63.4) | 84 (77.8) |  | Reference |
| ***JAK1* c.991-27C>T + *JAK2* c.-1132G>T + *STAT3* c.*1671T>C + *STAT3* c.-1937C>G** |  |  |  |  |
| CC + GG + TT + CC | 20 (38.5) | 12 (19.0) | **0.01** | 3.67 (1.25-10.79) |
| CT or TT + GT or TT + TC or CC + CG or GG | 32 (61.5) | 51 (81.0) |  | Reference |

N: number of cases; %: percentage; *OR: odds ratio adjusted by age, nevi and sun exposure by multivariate analysis; CI: confidence interval; NC: not calculated. Significant values are presented in bold

**Supplementary Table 6**. *JAK1*, *JAK2* and *STAT3* genotypes in 237 cutaneous melanoma patients stratified by clinical aspects

| **Genotype** | **Age** | | **Gender** | | **Phototype** | | **Nevi** | | **Sun exposure** | | **Sunburn episode** | | | **Type sun exposure** | |
| --- | --- | --- | --- | --- | --- | --- | --- | --- | --- | --- | --- | --- | --- | --- | --- |
|  | **≤ 55** | **> 55** | **Male** | **Female** | **I-II** | **III-IV** | **<20** | **≥ 20** | **Yes** | **No** | **Yes** | **No** | | **N/Int** | **Chronic** |
|  | **N (%)** | **N (%)** | **N (%)** | **N (%)** | **N (%)** | **N (%)** | **N (%)** | **N (%)** | **N (%)** | **N (%)** | **N (%)** | **N (%)** | | **N (%)** | **N (%)** |
| ***JAK1*c.1648+1272G>A** |  | |  | |  | |  | |  | |  | | |  | |
| GG | 48(38.4) | 51(41.5) | 58(45.3) | 41(34.2) | 63(40.1) | 32(43.2) | 38(40.4) | 59(39.9) | 82(41.8) | 15(36.6) | 55(41.7) | 38(40.9) | | 39(40.2) | 54(44.6) |
| GA or AA | 77(61.6) | 72(58.5) | 70(54.7) | 79 (65.8) | 94(59.9) | 42(56.8) | 56(59.6) | 89(60.1) | 114(58.2) | 26 (63.4) | 77(58.3) | 55(59.1) | | 58(59.8) | 67 (55.4) |
| *p-*value | 0.62 | | 0.07 | | 0.65 | | 0.93 | | 0.53 | | 0.9 | | | 0.51 | |
| GG or GA | 102(81.6) | 103(83.7) | 105(82.0) | 100(83.3) | 128(81.5) | 63(85.1) | 78(83.0) | 122(82.4) | 166(84.7) | 32 (78.0) | 113(85.6) | 76(81.7) | | 79(81.4) | 105(86.8) |
| AA | 23(18.4) | 20(16.3) | 23(18.0) | 20(16.7) | 29(18.5) | 11(14.9) | 16(17.0) | 26 (17.6) | 30(15.3) | 09(22.0) | 19(14.4) | 17(18.3) | | 18(18.6) | 16(13.2) |
| *p* value | 0.65 | | 0.78 | | 0.49 | | 0.91 | | 0.35 | | 0.43 | | | 0.28 | |
| ***JAK1* c.991-27C>T** |  | |  | |  | |  | |  | |  | | |  | |
| CC | 60(48.0) | 62(50.4) | 74(57.8) | 48(40.0) | 79(50.3) | 38(51.4) | 47(50.0) | 72(48.6) | 102(52.0) | 18(43.9) | 65(49.2) | 49(52.7) | | 46 47.4) | 67(55.4) |
| CT or TT | 65(52.0) | 61(49.6) | 54(42.2) | 72(60.0) | 78(49.7) | 36(48.6) | 47(50.0) | 76(51.4) | 94(48.0) | 23(56.1) | 67(50.8) | 44(47.3) | | 51(42.6) | 54(44.6) |
| *p-*value | 0.7 | | **0.005*** | | 0.88 | | 0.83 | | 0.34 | | 0.61 | | | 0.24 | |
| CC or CT | 109(87.2) | 111(90.2) | 113(88.3) | 107(89.2) | 136(86.6) | 69(93.2) | 83(88.3) | 131(88.5) | 174(88.8) | 36(87.8) | 120(90.9) | 81(87.1) | | 88(90.7) | 107(88.4) |
| TT | 16(12.8) | 12(9.8) | 15(11.7) | 13(10.8) | 21(13.4) | 05(6.8) | 11(11.7) | 17(11.5) | 22(11.2) | 05(12.2) | 12(9.1) | 12(12.9) | | 09(9.3) | 14(11.6) |
| *p-*value | 0.44 | | 0.82 | | 0.18 | | 0.95 | | 0.79 | | 0.36 | | | 0.66 | |
| ***JAK2* c.-1132G>T** |  | |  | |  | |  | |  |  | | |  | | |
| GG | 53(42.4) | 56(45.5) | 53(41.4) | 56 (46.7) | 78(49.7) | 27(36.5) | 43(45.7) | 65(43.9) | 92(46.9) | 14(34.1) | 53(40.2) | 50(53.8) | | 44(45.4) | 56(46.3) |
| GT or TT | 72(57.6) | 67(54.5) | 75(58.6) | 64(53.3) | 79(50.3) | 47(63.5) | 51(54.3) | 83(56.1) | 104(53.1) | 27(65.9) | 79(59.8) | 43(46.2) | | 53(54.6) | 65(53.7) |
| *p-*value | 0.62 | | 0.4 | | 0.06 | | 0.78 | | 0.13 | | 0.04 | | | 0.89 | |
| GG or GT | 106(84.8) | 109(88.6) | 109(85.2) | 106(88.3) | 137(87.3) | 65(87.8) | 80(85.1) | 132(89.2) | 170(86.7) | 36(87.8) | 118(89.4) | 80(86.0) | | 84(86.6) | 107(88.4) |
| TT | 19(15.2) | 14(11.4) | 19(14.8) | 14(11.7) | 20(12.7) | 09(12.2) | 14(14.9) | 16(10.8) | 26(13.3) | 05(12.2) | 14(10.6) | 13(14.0) | | 13(13.4) | 14(11.6) |
| *p* value | 0.37 | | 0.46 | | 1 | | 0.34 | | 1 | | 0.44 | | | 0.68 | |
| ***JAK2* c.-139G>A** |  | |  | |  | |  | |  |  | | |  | | |
| GG | 123(98.4) | 121(98.4) | 126(98.4) | 118(98.3) | 156(99.4) | 71(95.9) | 93(98.9) | 145(98.0) | 192(98.0) | 41(100.0) | 130(98.5) | 91(97.8) | | 96(99.0) | 118(97.5) |
| GA or AA | 02(1.6) | 02(1.6) | 02(1.6) | 02(1.7) | 01(0.6) | 03(4.1) | 01(1.1) | 03(2.0) | 04(2.0) | 0(0.0) | 02(1.5) | 02(2.2) | | 01(1.0) | 03(2.5) |
| *p-*value | 1 | | 1 | | 0.09 | | 1 | | 1 | | 1 | | | 0.63 | |
| GG or GA | 125(100.0) | 123(100.0) | 128(100.0) | 120(100.0) | 157(100.0) | 74(100.0) | 94(100.0) | 148(100.0) | 196(100.0) | 41(100.0) | 132(100.0) | 93(100.0) | | 97(100.0) | 121(100.0) |
| AA | 00(0.0) | 00(0.0) | 00(0.0) | 00(0.0) | 00(0.0) | 00(0.0) | 00(0.0) | 00(0.0) | 00(0.0) | 00(0.0) | 00(0.0) | 00(0.0) | | 00(0.0) | 00(0.0) |
| *p-*value | NC | | NC | | NC | | NC | | NC | | NC | | | NC | |
| ***STAT3* c.*1671T>C** |  | |  | |  | |  | |  |  | | |  | | |
| TT | 42(33.6) | 47(38.2) | 46(35.9) | 43(35.8) | 60(38.2) | 24(32.4) | 39(41.5) | 48(32.4) | 72(36.7) | 14(34.1) | 42(31.8) | 38(40.9) | | 34(35.1) | 46(38.0) |
| TC or CC | 83(66.4) | 76(61.8) | 82(64.1) | 77(64.2) | 97(61.8) | 50(67.6) | 55(58.5) | 100(67.6) | 124(63.3) | 27(65.9) | 90(68.2) | 55(59.1) | | 63(64.9) | 75(62.0) |
| *p-*value | 0.44 | | 0.98 | | 0.39 | | 0.15 | | 0.75 | | 0.16 | | | 0.65 | |
| TT or TC | 103(82.4) | 102(82.9) | 112(87.5) | 93(77.5) | 136(86.6) | 59(79.7) | 76(80.9) | 124(83.8) | 167(85.2) | 31(75.6) | 113(85.6) | 76(81.7) | | 82(84.5) | 99(81.8) |
| CC | 22(17.6) | 21(17.1) | 16(12.5) | 27(22.5) | 21(13.4) | 15(20.3) | 18(19.1) | 24(16.2) | 29(14.8) | 10(24.4) | 19(14.4) | 17(18.3) | | 15(15.5) | 22(18.2) |
| *p-*value | 0.91 | | 0.03 | | 0.17 | | 0.55 | | 0.13 | | 0.43 | | | 0.59 | |
| ***STAT3* c.-1937C>G** |  | |  | |  | |  | |  |  | | |  | | |
| CC | 60(48.0) | 71(57.7) | 71(55.5) | 60(50.0) | 87(55.4) | 36(48.6) | 54(57.4) | 74(50.0) | 105(53.6) | 18(43.9) | 69(52.3) | 50(53.8) | | 43(44.3) | 73(60.3) |
| CG or GG | 65(52.0) | 52(42.3) | 57(44.5) | 60(50.0) | 70(44.6) | 38(51.4) | 40(42.6) | 74(50.0) | 91(46.4) | 23(56.1) | 63(47.7) | 43(46.2) | | 54(55.7) | 48(39.7) |
| *p-*value | 0.12 | | 0.38 | | 0.33 | | 0.25 | | 0.26 | | 0.82 | | | 0.01 | |
| CC or CG | 112(89.6) | 114(92.7) | 119(93.0) | 107(89.2) | 149(94.9) | 63(85.1) | 86(91.5) | 135(91.2) | 183(93.4) | 33(80.5) | 122(92.4) | 83(89.2) | | 85(87.6) | 113(93.4) |
| GG | 13(10.4) | 09(7.3) | 09(7.0) | 13(10.8) | 08(5.1) | 11(14.9) | 08(8.5) | 13(8.8) | 13(6.6) | 08(19.5) | 10(7.6) | 10(10.8) | | 12(12.4) | 08(6.6) |
| *p-*value | 0.5 | | 0.37 | | 0.01 | | 1 | | 0.01 | | 0.41 | | | 0.16 | |

N: number of cases; %: percentage, Int: intermittent; NC: not calculated. Significant values are presented in bold. *: Significant even after Bonferroni correction for multiple comparisons (corrected *p* value = 0.007)

**Supplementary Table 7**. *JAK1*, *JAK2* and *STAT3* genotypes in 237 cutaneous melanoma patients stratified by pathological aspects

| **Genotypes** | **Tumor location** | | **Ulceration** | | **Type of growth** | | **Clark level** | | **Breslow (mm)** | | **Clinical stage** | | **Metastasis** | |
| --- | --- | --- | --- | --- | --- | --- | --- | --- | --- | --- | --- | --- | --- | --- |
|  | **Limbs** | **Axial** | **Yes** | **No** | **Vert** | **Hor** | **I+II** | **III-V** | **≤1.5** | **>1.5** | **0-II** | **III+IV** | **Yes** | **No** |
|  | **N (%)** | **N (%)** | **N (%)** | **N (%)** | **N (%)** | **N (%)** | **N (%)** | **N (%)** | **N (%)** | **N (%)** | **N (%)** | **N (%)** | **N (%)** | **N (%)** |
| ***JAK1*c.1648+1272G>A** |  | | | | | |  |  | | | | | | |
| GG | 67(40.1) | 32(39.5) | 33(48.5) | 37(34.6) | 47(42.3) | 12(36.4) | 35(33.8) | 65(41.7) | 41(34.7) | 46(45.1) | 67(40.9) | 21(37.5) | 69(37.7) | 08(44.4) |
| GA or AA | 100(59.9) | 49(60.5) | 35(51.5) | 70(65.4) | 64(57.7) | 21(63.6) | 49(66.2) | 91(58.3) | 77(65.3) | 56(54.9) | 97(59.1) | 35(62.5) | 114(62.3) | 10(55.6) |
| *p-*value | 0.92 | | 0.06 | | 0.54 | | 0.25 | | 0.11 | | 0.65 | | 0.61 | |
| GG or GA | 139(83.2) | 66(81.5) | 56(82.4) | 83(77.6) | 91(82.0) | 25(75.8) | 60(81.1) | 128(82.1) | 95(80.5) | 87(85.3) | 134(81.7) | 45(80.4) | 148(80.9) | 16(88.9) |
| AA | 28 (16.8) | 15(18.5) | 12(17.6) | 24(22.4) | 20(18.0) | 08(24.2) | 14(18.9) | 28(17.9) | 23(19.5) | 15(14.7) | 30(18.3) | 11(19.6) | 35(19.1) | 02(11.1) |
| *p-*value | 0.73 | | 0.44 | | 0.45 | | 0.85 | | 0.34 | | 0.82 | | 0.53 | |
| ***JAK1* c.991-27C>T** |  | | | | | |  |  | | | | | | |
| CC | 85(50.9) | 37(45.7) | 37(54.4) | 48(44.9) | 59(53.2) | 13(39.4) | 32(43.2) | 80(51.3) | 54(45.8) | 54(52.9) | 81(49.4) | 27(48.2) | 85(46.4) | 16(88.9) |
| CT or TT | 82(49.1) | 44(54.3) | 31(45.6) | 59(55.1) | 52(46.8) | 20(60.6) | 42(56.8) | 76(48.7) | 64(54.2) | 48(47.1) | 83(50.6) | 29(51.8) | 98(53.6) | 02(11.1) |
| *p-*value | 0.44 | | 0.21 | | 0.16 | | 0.25 | | 0.28 | | 0.87 | | 0.13 | |
| CC or CT | 150(89.8) | 70(86.4) | 61(89.7) | 90(84.1) | 99(89.2) | 27(81.8) | 64(86.5) | 139(89.1) | 103(87.3) | 93(91.2) | 145(88.4) | 49(87.5) | 161(88.0) | 17(94.4) |
| TT | 17(10.2) | 11(13.6) | 07(10.3) | 17(15.9) | 12(10.8) | 06(18.2) | 10(13.5) | 17(10.9) | 15(12.7) | 09(8.8) | 19(11.6) | 07(12.5) | 22(12.0) | 01(5.6) |
| *p-*value | 0.42 | | 0.37 | | 0.36 | | 0.56 | | 0.39 | | 0.81 | | 0.70 | |
| ***JAK2* c.-1132G>T** |  | | | | | |  |  | | | | | | |
| GG | 66(39.5) | 43(53.1) | 30(44.1) | 47(43.9) | 47(42.3) | 14(42.4) | 35(47.3) | 66(42.3) | 48(40.7) | 48(47.1) | 66(40.2) | 28(50.0) | 74(40.4) | 12(66.7) |
| GT or TT | 101(60.5) | 38(46.9) | 38(55.9) | 60(56.1) | 64(57.7) | 19(57.6) | 39(52.7) | 90(57.7) | 70(59.3) | 54(52.9) | 98(59.8) | 28(50.0) | 109(59.6) | 06(33.3) |
| *p-*value | **0.04*** | | 0.98 | | 0.99 | | 0.47 | | 0.34 | | 0.20 | | **0.04*** | |
| GG or GT | 139(83.2) | 76(93.8) | 61(89.7) | 93(86.9) | 99(89.2) | 28(84.8) | 63(85.1) | 138(88.5) | 102(86.4) | 91(89.2) | 143(87.2) | 47(83.9) | 160(87.4) | 16(88.9) |
| TT | 28(16.8) | 05(6.2) | 07(10.3) | 14(13.1) | 12(10.8) | 05(15.2) | 11(14.9) | 18(11.5) | 16(13.6) | 11(10.8) | 21(12.8) | 09(16.1) | 23(12.6) | 02(11.1) |
| *p-*value | **0.02*** | | 0.64 | | 0.54 | | 0.47 | | 0.53 | | 0.50 | | 1.00 | |
| ***JAK2* c.-139G>A** |  | | | | | |  |  | | | | | | |
| GG | 165(98.8) | 79(97.5) | 68(100) | 104(97.2) | 109(98.2) | 32(97.0) | 72(97.3) | 154(98.7) | 115(97.5) | 101(99.0) | 161(98.2) | 56(100.0) | 180(98.4) | 18(100) |
| GA or AA | 02(1.2) | 02(2.5) | 0(0.0) | 03(2.8) | 02(1.8) | 01(3.0) | 02(2.7) | 02(1.3) | 03(2.5) | 01(1.0) | 03(1.8) | 0(0.0) | 03(1.6) | 0(0.0) |
| *p-*value | 0.59 | | 0.28 | | 0.54 | | 0.59 | | 0.62 | | 0.57 | | 1.00 | |
| GG or GA | 167 (100) | 81(100) | 68(100) | 107(100) | 11 (100) | 33(100.0) | 74(100) | 156(100) | 118(100) | 102(100) | 164(100) | 56(100) | 183(100) | 18(100) |
| AA | 0(0.0) | 0(0.0) | 0(0.0) | 0(0.0) | 0(0.0) | 0(0.0) | 0(0.0) | 0(0.0) | 0(0.0) | 0(0.0) | 0(0.0) | 0(0.0) | 0(0.0) | 0(0.0) |
| *p-*value | NC | | NC | | NC | | NC | | NC | | NC | | NC | |
| ***STAT3* c.*1671T>C** |  | | | | | |  |  | | | | | | |
| TT | 62(37.1) | 27(33.3) | 31(45.6) | 37(34.6) | 44(39.6) | 14(42.4) | 24(32.4) | 58(37.2) | 43(36.4) | 35(34.3) | 55(33.5) | 22(39.3) | 66(36.1) | 07(38.9) |
| TC or CC | 105(62.9) | 54(66.7) | 37(54.4) | 70(65.4) | 67(60.4) | 19(57.6) | 50(67.6) | 98(62.8) | 75(63.6) | 67(65.7) | 109(66.5) | 34(60.7) | 117(63.9) | 11(61.1) |
| *p-*value | 0.55 | | 0.14 | | 0.77 | | 0.48 | | 0.74 | | 0.43 | | 0.80 | |
| TT or TC | 142(85.0) | 63(77.8) | 61(89.7) | 83(77.6) | 92(82.9) | 25(75.8) | 57(77.0) | 131(84.0) | 94(79.7) | 88(86.3) | 136(82.9) | 47(83.9) | 150(82.0) | 18(100) |
| CC | 25(15.0) | 18(22.2) | 07(10.3) | 24(22.4) | 19(17.1) | 08(24.2) | 17(23.0) | 25(16.0) | 24(20.3) | 14(13.7) | 28(17.1) | 09(16.1) | 33(18.0) | 0(0.0) |
| *p* value | 0.15 | | **0.04*** | | 0.44 | | 0.20 | | 0.19 | | 1.00 | | **0.04*** | |
| ***STAT3* c.-1937C>G** |  | | | | | |  |  | | | | | | |
| CC | 93(55.7) | 38(46.9) | 42(61.8) | 50(46.7) | 64(57.7) | 16(48.5) | 33(44.6) | 84(53.8) | 62(52.5) | 52(51.0) | 86(52.4) | 29(51.8) | 94(51.4) | 12(66.7) |
| CG or GG | 74(44.3) | 43(53.1) | 26(38.2) | 57(53.3) | 47(42.3) | 17(51.5) | 41(55.4) | 72(46.2) | 56(47.5) | 50(49.0) | 78(47.6) | 27(48.2) | 89(48.6) | 06(33.3) |
| *p-*value | 0.19 | | **0.05** | | 0.35 | | 0.19 | | 0.81 | | 0.93 | | 0.32 | |
| CC or CG | 153(91.6) | 73(90.1) | 64(94.1) | 99(92.5) | 103(92.8) | 30(90.9) | 67(90.5) | 143(91.7) | 108(91.5) | 94(92.2) | 154(93.9) | 47(83.9) | 166(90.7) | 17(94.4) |
| GG | 14(8.4) | 08(9.9) | 04(5.9) | 08(7.5) | 08(7.2) | 03(9.1) | 07(9.5) | 13(8.3) | 10(8.5) | 08(7.8) | 10(6.1) | 09(16.1) | 17(9.3) | 01(5.6) |
| *p-*value | 0.81 | | 0.76 | | 0.71 | | 0.80 | | 1.00 | | **0.02*** | | 1.00 | |

N: number of cases; %: percentage; Vert: vertical, Hor: horizontal; NC: not calculated. *: Non-significant after Bonferroni correction for multiple comparisons (corrected *p* value = 0.007)

**Supplementary Table 8**. Clinicopathological aspects, *JAK1*, *JAK2* and *STAT3* genotypes in survival of 237 cutaneous melanoma patients

| **Characteristic** | **Univariate analysis** | | | | | | **Multivariate analysis** | | | | |
| --- | --- | --- | --- | --- | --- | --- | --- | --- | --- | --- | --- |
|  | **N event/**  **N total** | **PFS HR (95% CI)** | ***p-*value** | **N event/ N total** | **MSS**  **HR (95% CI)** | ***p-*value** | **PSF adjusted HR (95% CI)** | | ***p-*value** | **MSS adjusted HR (95% CI)** | ***p-*value** |
| **Age (years)** |  |  |  |  |  |  |  | |  |  |  |
| ≤ 55 | 41/118 | Reference | 0.24 | 19/118 | Reference | **0.02** | NA | | | Reference | 0.15 |
| > 55 | 49/119 | 1.28 (0.84-1.93) |  | 33/119 | 1.87 (1.06-3.29) |  |  |  |  | 1.62 (0.83-3.14) |  |
| **Gender** |  |  |  |  |  |  |  | |  |  |  |
| Male | 57/123 | 1.78 (1.16-2.73) | **0.008** | 39/123 | 3.17 (1.69-5.94) | **< 0.0001** | 1.12 (0.58-2.17) | | 0.72 | 1.84 (0.90-3.76) | 0.09 |
| Female | 33/114 | Reference |  | 13/114 | Reference |  | Reference | |  | Reference |  |
| **Tumor location** |  |  |  |  |  |  |  | |  |  |  |
| Limbs | 22/75 | Reference | **0.05** | 8/75 | Reference | **0.007** | Reference | **0.03** | | Reference | **0.005** |
| Axial | 68/162 | 1.59 (1.00-2.58) |  | 44/162 | 2.80 (1.32-5.96) |  | 2.42 (1.06-5.49) |  |  | 4.52 (1.56-13.10) |  |
| **Type of grown** |  |  |  |  |  |  |  | |  |  |  |
| Vertical | 41/108 | 2.89 (1.14-7.32) | **0.02** | 20/108 | 1.62 (0.55-4.74) | 0.37 | 3.30 (0.93-11.71) | 0.06 | | NA | |
| Horizontal | 05/33 | Reference |  | 4/33 | Reference |  | Reference |  | |  |  |
| **Clark levels** |  |  |  |  |  |  |  | |  |  |  |
| I or II | 8/73 | Reference | **< 0.0001** | 3/73 | Reference | **0.001** | Reference | | 0.09 | Reference | 0.55 |
| III to V | 71/150 | 5.51 (2.65-11.46) |  | 41/150 | 7.64 (2.36-24.68) |  | 3.90 (0.80-18.90) | |  | 1.63 (0.32-8.28) |  |
| **Breslow thickness (mm)** |  |  |  |  |  |  |  | |  |  |  |
| ≤ 1.5 | 17/116 | Reference | **< 0.0001** | 4/116 | Reference | **< 0.0001** | Reference | | **0.001** | Reference | **< 0.0001** |
| > 1.5 | 60/98 | 6.35 (3.69-10.91) |  | 37/98 | 13.48 (4.80-37.85) |  | 4.26 (1.82-9.97) | |  | 7.66 (2.61-22.40) |  |
| **Tumor stage** |  |  |  |  |  |  |  | |  |  |  |
| 0 to III | 41/163 | Reference | **< 0.0001** | 18/163 | Reference | **< 0.0001** | Reference | | **< 0.0001** | Reference | **< 0.0001** |
| III or IV | 41/48 | 6.43 (4.10-10.09) |  | 30/48 | 8.24 (4.58-14.83) |  | 4.08 (2.04-8.15) | |  | 3.95 (2.03-7.69) |  |
| ***JAK1* c.1648+1272G>A** |  |  |  |  |  |  |  | |  |  |  |
| GG | 36/93 | Reference | 0.70 | 23/93 | 1.29 (0.74-2.23) | 0.35 | NA | | | NA |  |
| GA or AA | 54/144 | 1.08 (0.71-1.65) |  | 29/144 | Reference |  |  |  |  |  |  |
| GG or GA | 73/196 | Reference | 0.62 | 41/196 | 1.26 (0.65-2.46) | 0.48 | NA | | | NA | |
| AA | 17/41 | 1.14 (0.67-1.93) |  | 11/41 | Reference |  |  |  |  |  |  |
| ***JAK1* c.991-27C>T** |  |  |  |  |  |  |  | |  | NA |  |
| CC | 43/115 | Reference | 0.88 | 27/115 | 1.19 (0.69-2.06) | 0.51 | NA | | |  |  |
| CT or TT | 47/122 | 1.03 (0.68-1.56) |  | 25/122 | Reference |  |  |  |  |  |  |
| CC or CT | 79/210 | Reference | 0.85 | 45/210 | 1.20 (0.54-2.67) | 0.64 | NA | | | NA | |
| TT | 11/27 | 1.06 (0.56-2.00) |  | 7/27 | Reference |  |  |  |  |  |  |
| ***JAK2* c.-1132G>T** |  |  |  |  |  |  |  | |  |  |  |
| GG | 40/104 | Reference | 0.85 | 23/104 | Reference | 0.99 | NA | | | NA | |
| GT or TT | 50/133 | 1.03 (0.68-1.57) |  | 29/133 | 1.00 (0.57-1.73) |  |  |  |  |  |  |
| GG or GT | 78/206 | Reference | 0.82 | 45/206 | Reference | 0.79 | NA | | | NA | |
| TT | 12/31 | 1.07 (0.58-1.96) |  | 7/31 | 1.10 (0.50-2.46) |  |  |  |  |  |  |
| ***JAK2* c.-139G>A** |  |  |  |  |  |  |  | |  |  |  |
| GG | 89/233 | Reference | 0.59 | 51/233 | Reference | 0.90 | NA | | | NA | |
| GA or AA | 01/04 | 1.69 (0.23-12.19) |  | ¼ | 1.13 (0.15-8.18) |  |  |  |  |  |  |
| GG or GA | 90/237 | Reference | NC | 52/237 | Reference | NC | NA | | | NA | |
| AA | 0/0 | NC |  | 0/0 | NC |  |  |  |  |  |  |
| ***STAT3* c.*1671T>C** |  |  |  |  |  |  |  | |  |  |  |
| TT | 28/84 | 1.20 (0.76-1.87) | 0.41 | 18/84 | 1.04 (0.58-1.84) | 0.89 | NA | | | NA | |
| TC or CC | 62/153 | Reference |  | 34/153 | Reference |  |  |  |  |  |  |
| TT or TC | 76/197 | 1.18 (0.67-2.10) | 0.55 | 45/197 | 1.38 (0.62-3.06) | 0.42 | NA | | | NA | |
| CC | 14/40 | Reference |  | 7/40 | Reference |  |  |  |  |  |  |
| ***STAT3* c.-1937C>G** |  |  |  |  |  |  |  | |  |  |  |
| CC | 45/126 | 1.07 (0.71-1.62) | 0.73 | 27/126 | 1.01 (0.59-1.75) | 0.95 | NA | | | NA | |
| CG or GG | 45/111 | Reference |  | 25/111 | Reference |  |  |  |  |  |  |
| CC or CG | 80/216 | 1.16 (0.60-2.25) | 0.64 | 48/216 | 1.30 (0.46-3.61) | 0.61 | NA | | | NA | |
| GG | 10/21 | Reference |  | 4/21 | Reference |  |  |  |  |  |  |

N: number of individuals; PFS: progression‐free survival; MSS: melanoma-specific survival; HR: hazard ratio; CI: confidence interval; mm: millimeters; NA: not analyzed. Significant values are presented in bold

**Supplementary Table 9.** *JAK1, JAK2* and *STAT3* gene expression in samples of peripheral blood leukocytes from cutaneous melanoma patients

| **Genotype/Allele** | **N** | **Average (AU)** | **Median (AU)** | **SD (AU)** | **Minimum (AU)** | **Maximum (AU)** | ***p*-value** |
| --- | --- | --- | --- | --- | --- | --- | --- |
| ***JAK1*c.1648+1272G>A** | | | | | | | |
| GG | 18 | 1.27 | 1.59 | 0.65 | 0.12 | 2.26 | 0.46 |
| GA | 18 | 0.84 | 0.7 | 0.72 | 0.12 | 2.66 | 0.84 |
| AA | 6 | 0.93 | 0.59 | 0.73 | 0.15 | 1.95 | Reference |
| GG | 18 | 1.27 | 1.59 | 0.65 | 0.12 | 2.26 | 0.06 |
| GA or AA | 24 | 0.87 | 0.64 | 0.71 | 0.12 | 2.66 | Reference |
| GG or GA | 36 | 1.05 | 0.93 | 0.71 | 0.12 | 2.66 | 0.79 |
| AA | 6 | 0.93 | 0.59 | 0.73 | 0.15 | 1.95 | Reference |
| Allele G | 54 | 1.12 | 1.11 | 0.69 | 0.12 | 2.66 | 0.12 |
| Allele A | 30 | 0.88 | 0.6 | 0.7 | 0.12 | 2.66 | Reference |
| ***JAK1* c.991-27C>T** | | | | | | | |
| CC | 22 | 1.22 | 1.53 | 0.6 | 0.11 | 2.2 | 0.07 |
| CT | 15 | 0.78 | 0.52 | 0.75 | 0.12 | 2.59 | 0.85 |
| TT | 3 | 0.57 | 0.57 | 0.01 | 0.55 | 0.59 | Reference |
| CC | 22 | 1.22 | 1.53 | 0.6 | 0.11 | 2.2 | **0.01** |
| CT or TT | 18 | 0.75 | 0.56 | 0.69 | 0.12 | 2.59 | Reference |
| CC or CT | 37 | 1.04 | 0.93 | 0.69 | 0.11 | 2.59 | 0.34 |
| TT | 3 | 0.57 | 0.57 | 0.01 | 0.55 | 0.59 | Reference |
| Allele C | 59 | 1.11 | 1.08 | 0.66 | 0.11 | 2.59 | **0.02** |
| AlleleT | 21 | 0.75 | 0.57 | 0.64 | 0.12 | 2.59 | Reference |
| ***JAK2* c.-1132G>T** | | | | | | | |
| GG | 13 | 1.18 | 1.19 | 0.62 | 0.22 | 2.35 | Reference |
| GT | 9 | 0.75 | 0.83 | 0.38 | 0.13 | 1.14 | 0.08 |
| TT | 8 | 1.25 | 1.2 | 0.61 | 0.5 | 2.41 | 0.81 |
| GG | 13 | 1.18 | 1.19 | 0.62 | 0.22 | 2.35 | Reference |
| GT or TT | 17 | 0.99 | 0.95 | 0.54 | 0.13 | 2.41 | 0.37 |
| GG or GT | 22 | 1.01 | 0.95 | 0.57 | 0.13 | 2.35 | Reference |
| TT | 8 | 1.25 | 1.2 | 0.61 | 0.5 | 2.41 | 0.32 |
| Allele G | 35 | 1.07 | 0.95 | 0.58 | 0.13 | 2.35 | Reference |
| Allele T | 25 | 1.07 | 1.1 | 0.57 | 0.13 | 2.41 | 0.99 |
| ***STAT3* c.*1671T>C** | | | | | | | |
| TT | 13 | 1.32 | 1.22 | 0.62 | 0.4 | 2.52 | Reference |
| TC | 17 | 1.04 | 1.13 | 0.49 | 0.28 | 1.73 | 0.18 |
| CC | 5 | 1.76 | 2.16 | 0.64 | 0.73 | 2.23 | 0.2 |
| TT | 13 | 1.32 | 1.22 | 0.62 | 0.4 | 2.52 | Reference |
| TC or CC | 22 | 1.2 | 1.22 | 0.6 | 0.28 | 2.23 | 0.59 |
| TT or TC | 30 | 1.16 | 1.18 | 0.56 | 0.28 | 2.52 | Reference |
| CC | 5 | 1.76 | 2.16 | 0.64 | 0.73 | 2.23 | **0.03** |
| Allele T | 43 | 1.21 | 1.21 | 0.58 | 0.28 | 2.52 | Reference |
| Allele C | 27 | 1.3 | 1.24 | 0.63 | 0.28 | 2.23 | 0.51 |
| ***STAT3* c.-1937C>G** | | | | | | | |
| CC | 24 | 1.3 | 1.18 | 0.83 | 0.11 | 3.12 | Reference |
| CG | 13 | 1.24 | 1.25 | 0.67 | 0.28 | 2.25 | 0.82 |
| GG | 3 | 1.65 | 1.53 | 0.47 | 1.24 | 2.18 | 0.49 |
| CC | 24 | 1.3 | 1.18 | 0.83 | 0.11 | 3.12 | Reference |
| CG or GG | 16 | 1.32 | 1.39 | 0.65 | 0.28 | 2.25 | 0.95 |
| CC or CG | 37 | 1.28 | 1.22 | 0.77 | 0.11 | 3.12 | Reference |
| GG | 3 | 1.65 | 1.53 | 0.47 | 1.24 | 2.18 | 0.42 |
| Allele C | 61 | 1.29 | 1.22 | 0.79 | 0.11 | 3.12 | Reference |
| Allele G | 19 | 1.37 | 1.53 | 0.62 | 0.28 | 2.25 | 0.44 |

N: number of cases; SD: standard deviation; AU: arbitrary unit. Significant values are presented in bold
